# Supplementary material for: Small molecule SWELL1 complex induction improves glycemic control and nonalcoholic fatty liver disease in murine Type 2 diabetes
Source: Nat Commun. 2022 Feb 10;13:784. doi: 10.1038/s41467-022-28435-0 (PMC8831520; doi:10.1038/s41467-022-28435-0)
Supplement: Supplementary file 1 — Supplementary Information [file 41467_2022_28435_MOESM1_ESM.pdf]

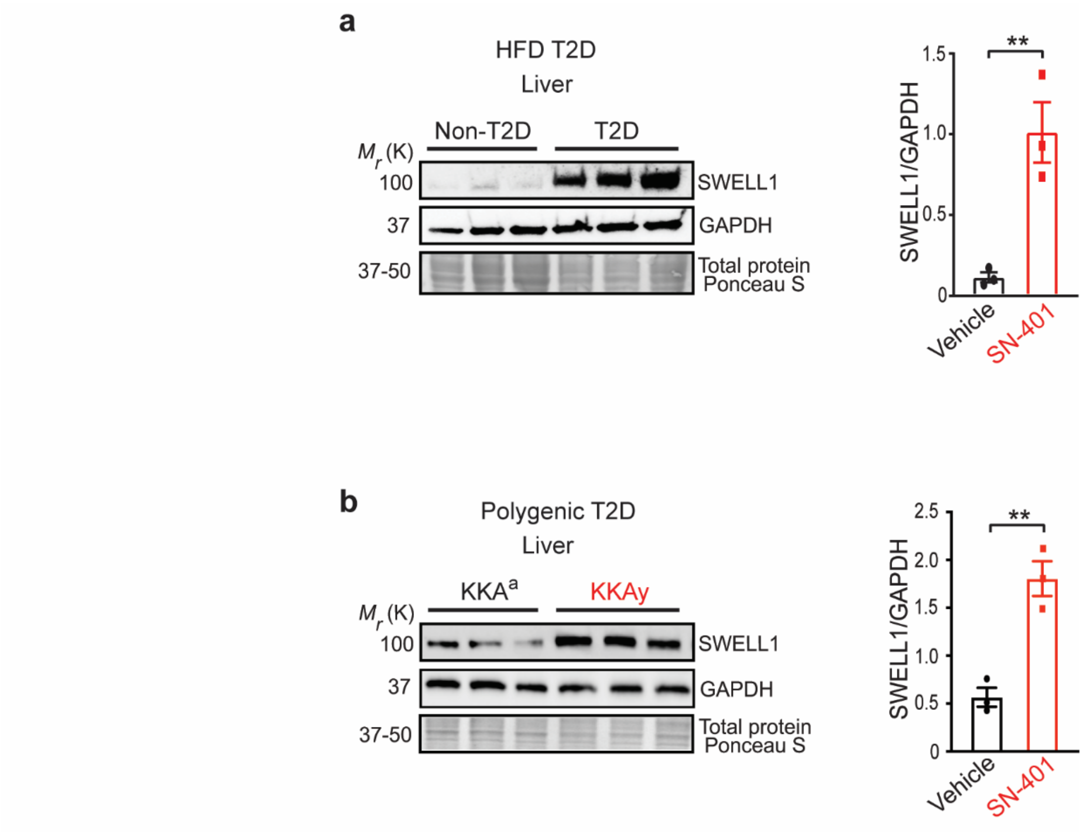

Supplementary Fig. S1. Hepatic SWELL1 protein expression in high-fat diet (HFD) and polygenic-T2D mice models. a-b. Western blot for SWELL1 protein expression in liver tissue isolated from C57 HFD (33 weeks HFD) (**a**) and polygenic-T2D KKA^y (30-40 weeks old) (**b**) mice compared to their respective controls (n=3 each). Data are represented as mean ±SEM. Two-tailed unpaired t-test was used in **a** & **b.** *, ** and *** represents *p<0.05*, *p<0.01* and *p<0.001* respectively.

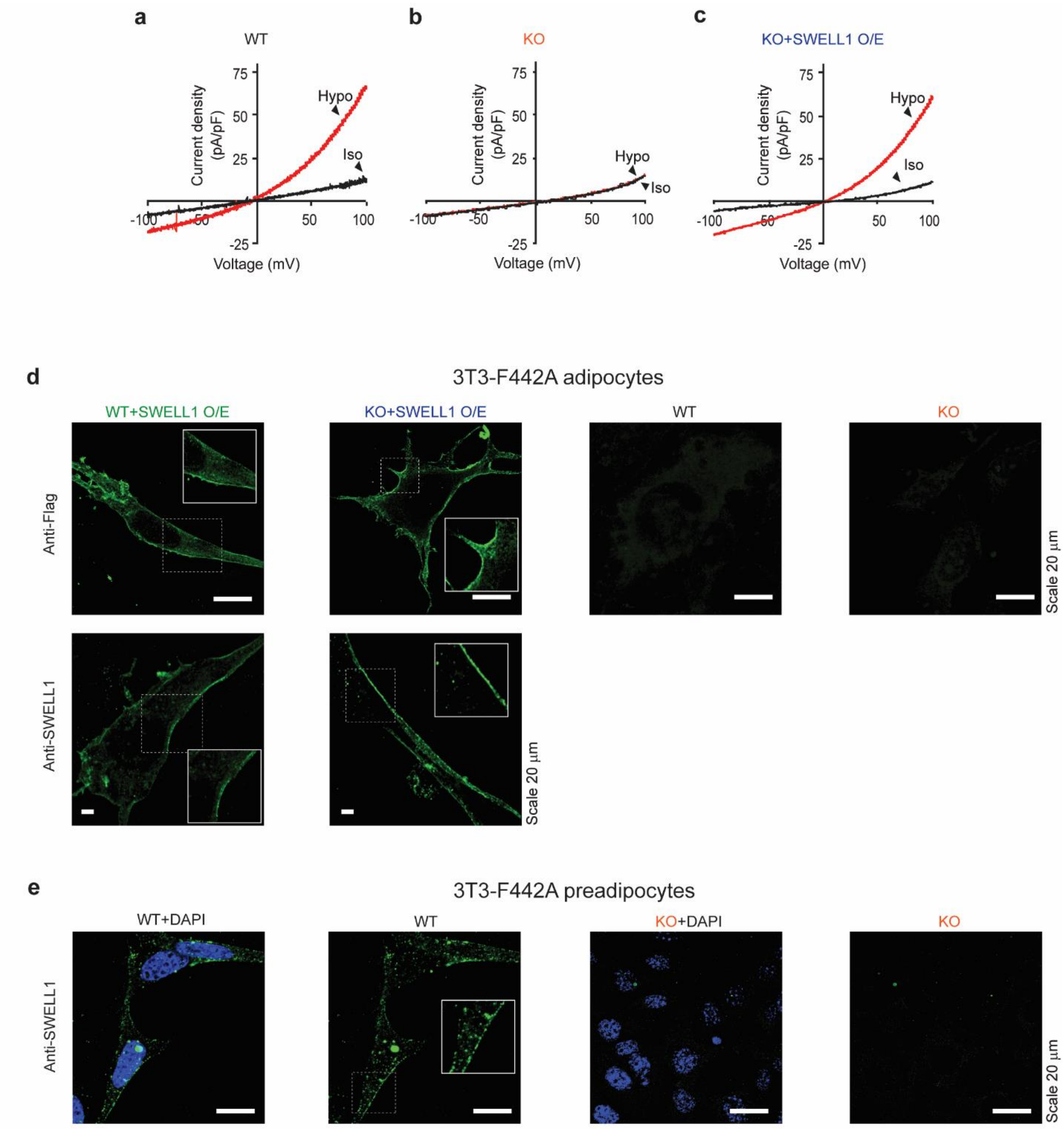

Supplementary Fig. S2. Transient expression of full-length SWELL1 with C-terminal 3XFlag tag rescues $I_{Cl,SWELL}$ and traffics to the plasma membrane.

a-c. Current-voltage plots of $I_{Cl,SWELL}$ measured in 3T3-F442A preadipocytes WT **(a)**, KO **(b)** and adenoviral re-expression of SWELL1 in KO (KO+SWELL1 O/E) **(c)** at baseline (iso, black trace) and hypotonic (hypo, red trace) stimulation respectively. **d.** Immunostaining images

demonstrating localization of endogenous SWELL1 or overexpressed SWELL1 with anti-Flag or anti-SWELL1 antibody (Scale bar: 20 µm) in 3T3-F442A adipocytes. **e.** Validation of SWELL1 antibody in WT compared to SWELL1 KO 3T3-F442A pre-adipocytes (Scale bar – 20 µm), revealing a punctate pattern of endogenous SWELL1 localization (inset).

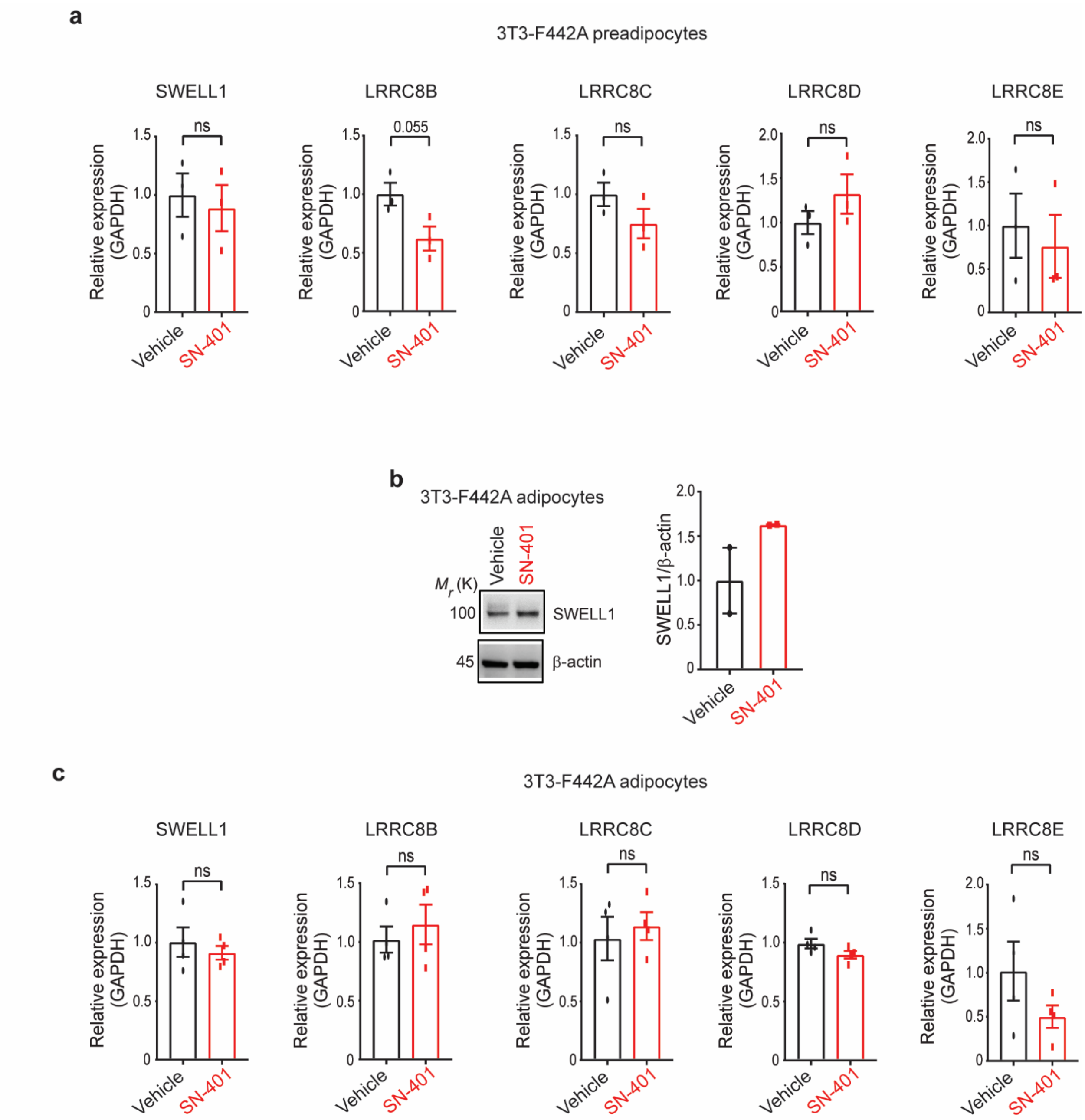

Supplementary Fig. S3. Increases in SWELL1 protein are not associated with increases in mRNA expression of SWELL1/LRRC8a or LRRC8b-e. a. Relative mRNA expression of LRRC8 family members to GAPDH assessed by qPCR (n = 3 each) for 3T3 F-442A preadipocytes treated with either vehicle or 10 µM SN-401 for 96 hours. **b.** Western blots showing increase in SWELL1 protein expression relative to β-actin in 3T3-F442A adipocytes treated with either vehicle or 10 µM SN-401 for 96 hours and the corresponding densitometric

ratio (n=2 each). **c**. Relative mRNA expression of LRRC8 family members relative to GAPDH assessed by qPCR (n = 4 each) from the same 3T3-F442A adipocytes treated with either vehicle or 10 µM SN-401 for 96 hours obtained from **b**. Data are represented as mean ±SEM. Two-tailed unpaired t-test was used in **a** and **c** where *, ** and *** represents $p<0.05$, $p<0.01$ and $p<0.001$ respectively.

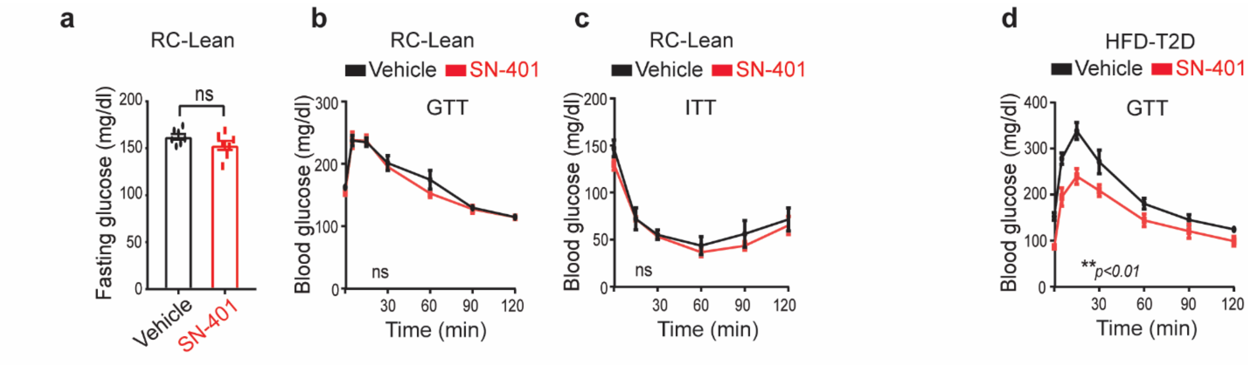

Supplementary Fig. S4. SN-401 activity in lean non-T2D mice and effects of chronic dosing in HFD-fed mice. a-c. Fasting glucose levels **(a)**, GTT **(b)** and ITT **(c)** of C57BL/6 lean mice on regular-chow diet treated with either vehicle or SN-401 (5 mg/kg i.p) for 10 days (n = 7 males in each group). **d.** GTT of HFD-T2D mice (8 weeks HFD) treated with either vehicle (n = 5 males) or SN-401 (5 mg/kg i.p, n = 4 males) for 8 weeks. Data are represented as mean ±SEM. Two-tailed unpaired t-test was used in **a** for FG**.** Two-way ANOVA was used in **b-d**. Statistical significance is denoted by *, ** and *** representing *p<0.05*, *p<0.01* and *p<0.001* respectively 'ns' indicates the difference was not significant.

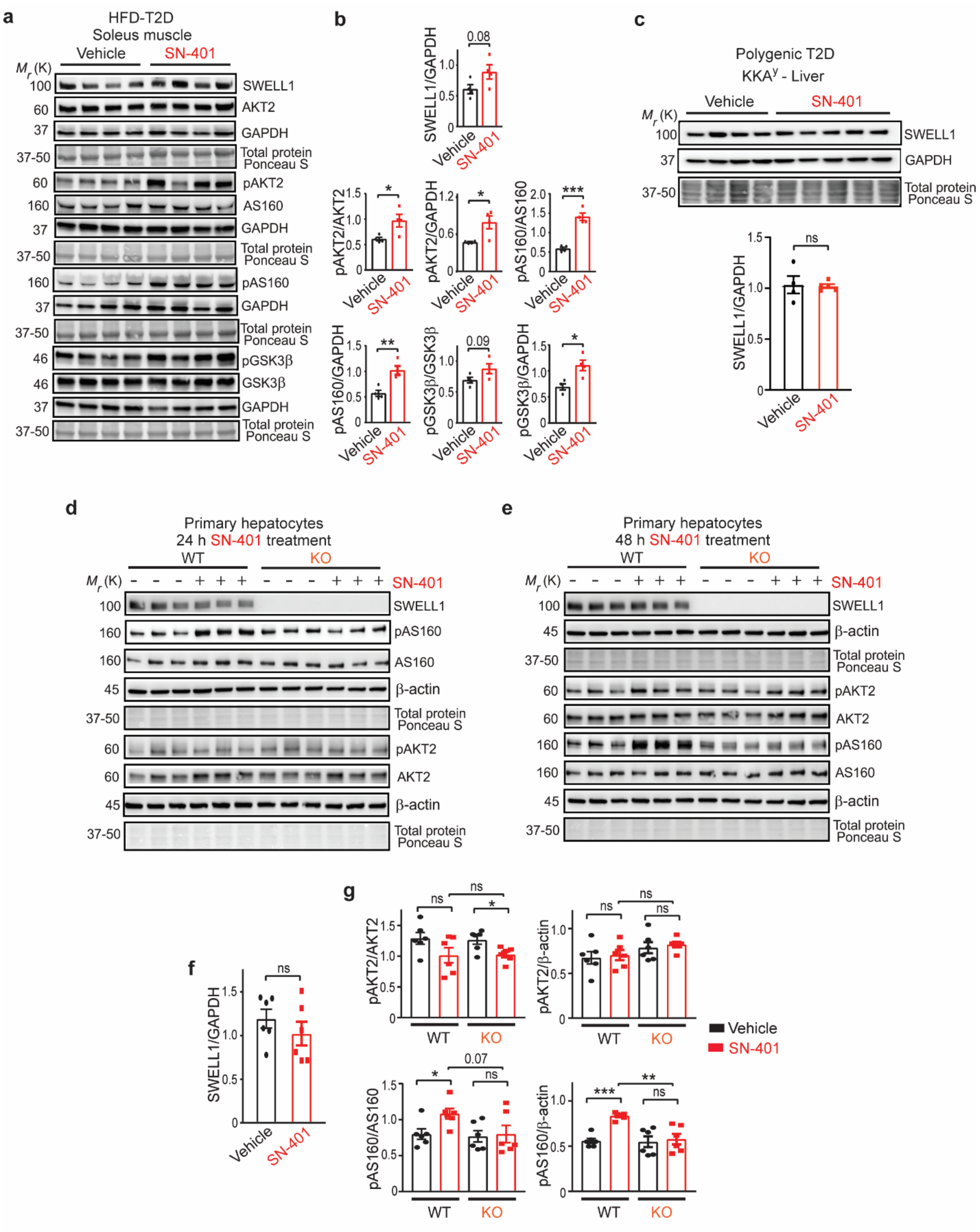

Supplementary Fig. S5. SN-401 augments SWELL1-AKT2-AS160-GSK3β in skeletal muscle and SWELL1 dependent AS160 signaling in hepatocytes. a-b. Western blots

detecting SWELL1, pAKT2^{Ser474}, AKT2, pAS160^{Thr642}, AS160, pGSK3β^{Ser9}, GSK3β and GAPDH in soleus muscle isolated from HFD-T2D (18 weeks HFD) treated with either vehicle or SN-401 (n = 4 each) for 4 days (5 mg/kg; i.p) (**a**) and the corresponding densitometric ratios (**b**). **c.** Western blots detecting SWELL1 and GAPDH in liver isolated from T2D KKA^{y} mice (12 weeks old) treated with either vehicle (n=4) or SN-401 (n = 5) for 5 days (5 mg/kg; i.p) and the corresponding densitometric ratio below. **d-g.** Western blots detecting SWELL1, pAKT2^{Ser474}, AKT2, pAS160^{Thr642}, AS160 and β-actin in primary hepatocytes isolated from SWELL1$^{fl/fl}$ (Wildtype, WT) and Albumin-Cre/SWELL1$^{fl/fl}$ (Knockout, KO) treated with either vehicle or 10 µM SN-401 (n = 3 each) for 24 h (**d**) or 48 h (**e**) and the corresponding densitometric ratios combined from both 24 and 48 hour treatment timepoints (**f,g**). Data are represented as mean ±SEM. Two-tailed unpaired t-test was used in **b, c, f** and **g** where *, ** and *** represents $p<0.05$, $p<0.01$ and $p<0.001$ respectively. 'ns' indicates the difference was not significant.

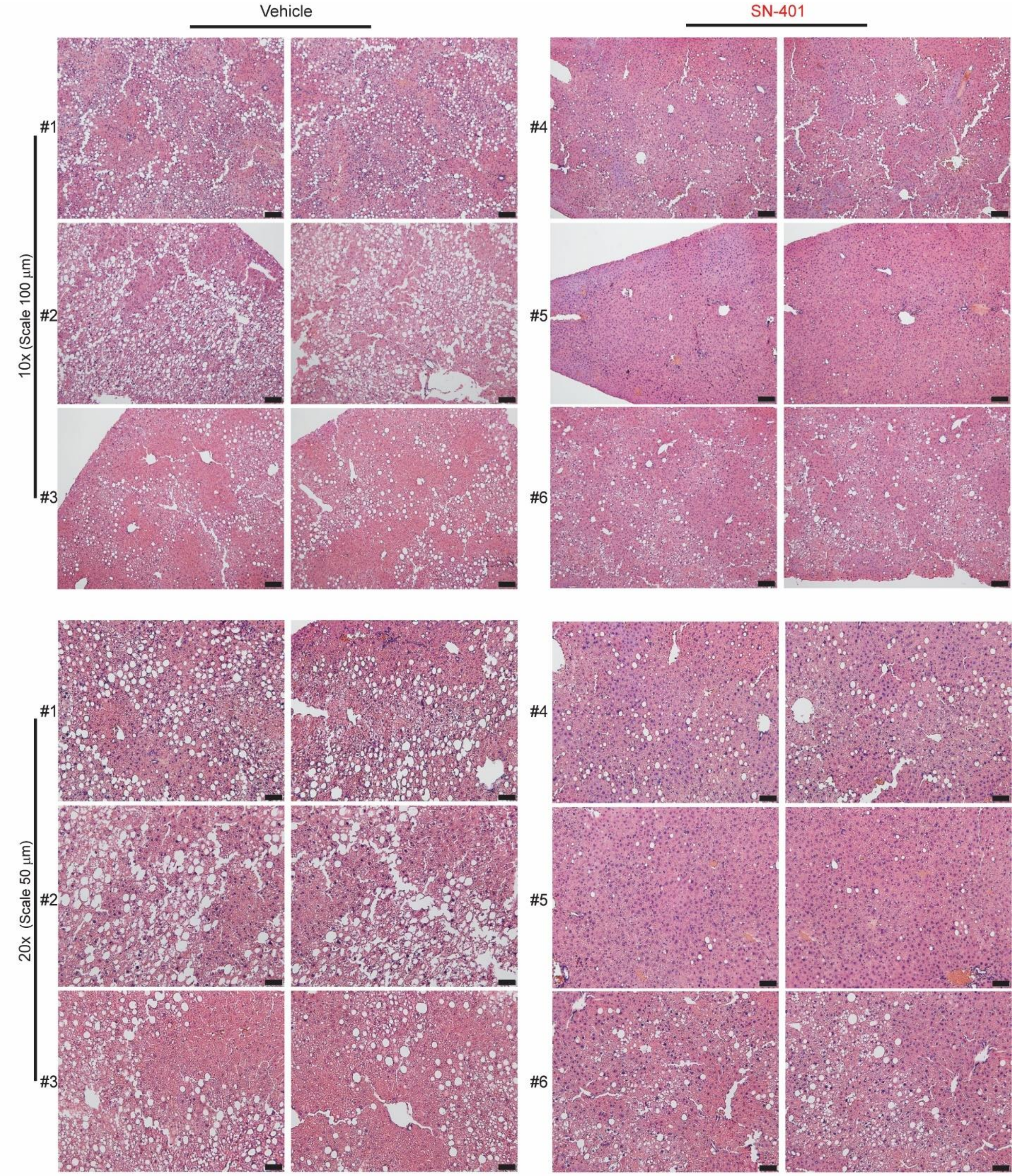

Supplementary Fig. S6. SN-401 improves non-alcoholic fatty liver disease in murine T2D models. Images of hematoxylin and eosin stained liver histology sections of HFD-T2D mice

treated with either vehicle or SN-401 (5 mg/kg i.p) as in **Figure. 4e**. Scale: 10X, 100 µm and 20X, 50 µm).

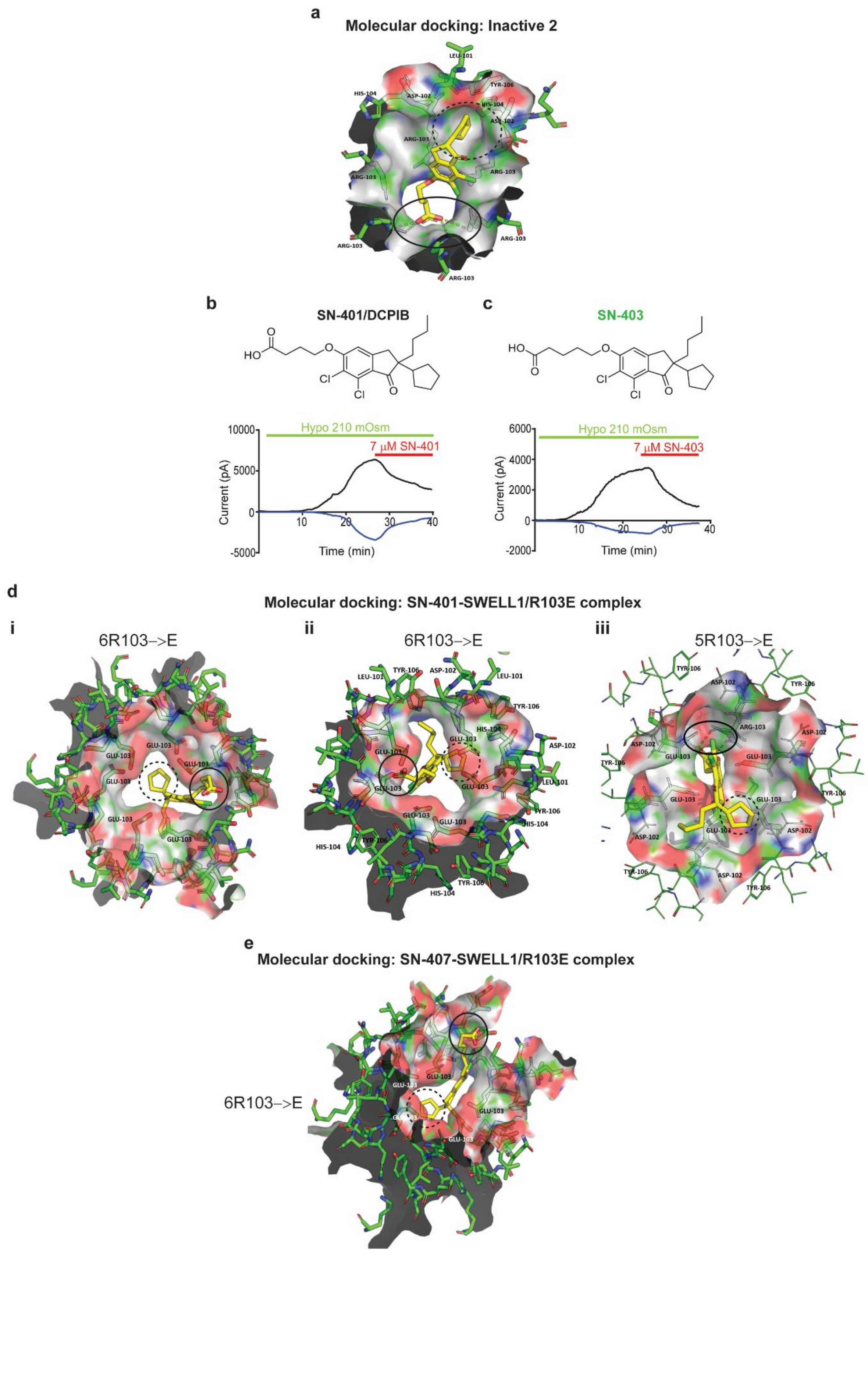
a
Molecular docking: Inactive 2
LEU-101
HIS-104
ASP-102
TYR-106
HIS-104
ASP-102
ARG-103
ARG-103
ARG-103
ARG-103
ARG-103
ARG-103
ARG-103
b
SN-401/DCPIB
c
SN-403
HO
O
Cl
Cl
Hypo 210 mOsm
7 μM SN-401
7 μM SN-403
Current (pA)
Time (min)
10000
5000
0
-5000
6000
4000
2000
-2000
10
20
30
40
d
Molecular docking: SN-401-SWELL1/R103E complex
i
6R103->E
ii
6R103->E
iii
5R103->E
GLU-103
LEU-101
TYR-106
ASP-102
HIS-104
ARG-103
e
Molecular docking: SN-407-SWELL1/R103E complex
6R103->E

Supplementary Fig. S7. Defining the SN-401-SWELL1 structure-activity relationship by combining chemical synthesis, molecular docking simulations and patch-clamp electrophysiology. a. Binding pose for Inactive 2 reveal that the carboxylate group can reach and electrostatically interact with R103 (solid black circle) but in the absence of the butyl group cannot orient the cyclopentyl ring (black broken circle) to occupy the hydrophobic cleft without introducing excessive structural strain on the carbon connecting the core with the cyclopentyl ring. **b-c.** Chemical structures (top) of SN-401/DCPIB (**b**) and SN-403 (**c**) and $I_{Cl,SWELL}$ inward and outward current over time (bottom) upon hypotonic (210 mOsm) stimulation and subsequent inhibition by 7 μM SN-401 and SN-403 in HEK-293 cells. **d.** Binding poses for SN-401 within the R103E-SWELL1 mutant hexamer complex wherein six (**i**, **ii**) or five (**iii**) arginine (R103) residues have been mutated to glutamic acid (E103) at amino acid 103. Molecular docking simulations reveal that neither the carboxylate group electrostatically interact with R103 (solid black circle) nor the cyclopentyl ring (black broken circle) occupy the hydrophobic cleft in two different poses obtained (**i**, **ii**). When five R103s are mutated to E103, docking revealed very few poses that include R103 interactions with the carboxylate group of SN-401 and cyclopentyl ring located near the hydrophobic cleft (**iii**). **e.** Binding pose for SN-407 within the R103E-SWELL1 mutant hexamer complex wherein six arginine (R103) residues have been mutated to glutamic acid (E103). Molecular docking simulations reveal that neither the carboxylate group electrostatically interact with R103 (solid black circle) nor the cyclopentyl ring (black broken circle) occupy the hydrophobic cleft.

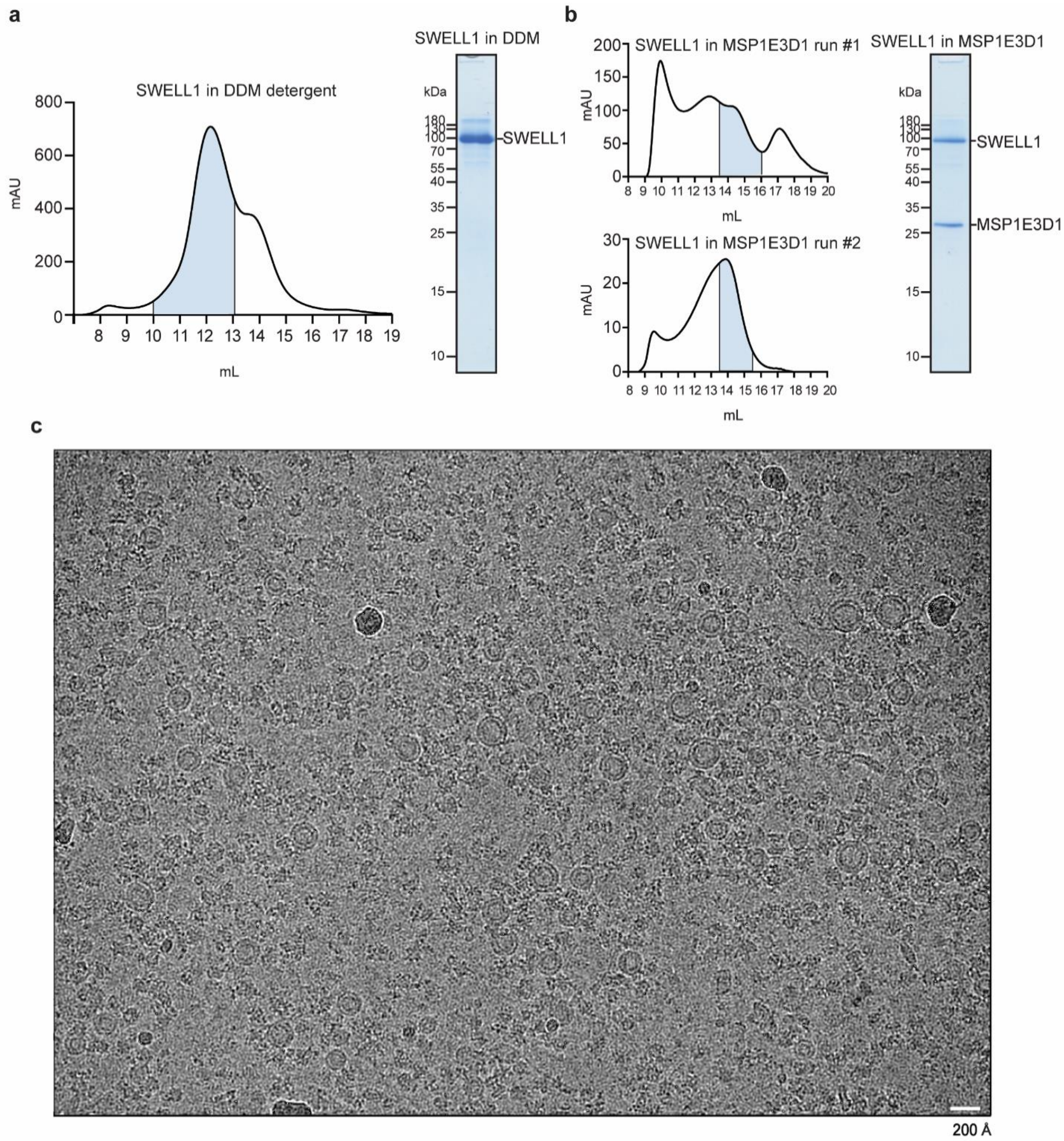

Supplementary Fig. S8. Purification, reconstitution, and cryo-EM imaging of SWELL1. a. Size exclusion chromatogram (Superose 6 Increase) of SWELL1 purified into DDM detergent (left). Pooled fractions corresponding to hexameric SWELL1 are highlighted in blue. Coomassie-stained SDS-PAGE of pooled SWELL1 homohexamer-containing fractions (right). **b.** Size exclusion chromatogram of SWELL1 reconstituted into MSP1E3D1 lipid nanodiscs (left-upper).

Pooled fractions were then re-run (left-lower) and pooled fractions were concentrated for drug addition, grid freezing, and coomassie-stained SDS-PAGE (right). **c.** Example micrograph from SN-407- SWELL1 in MSP1E3D1 cryo-EM data collection.

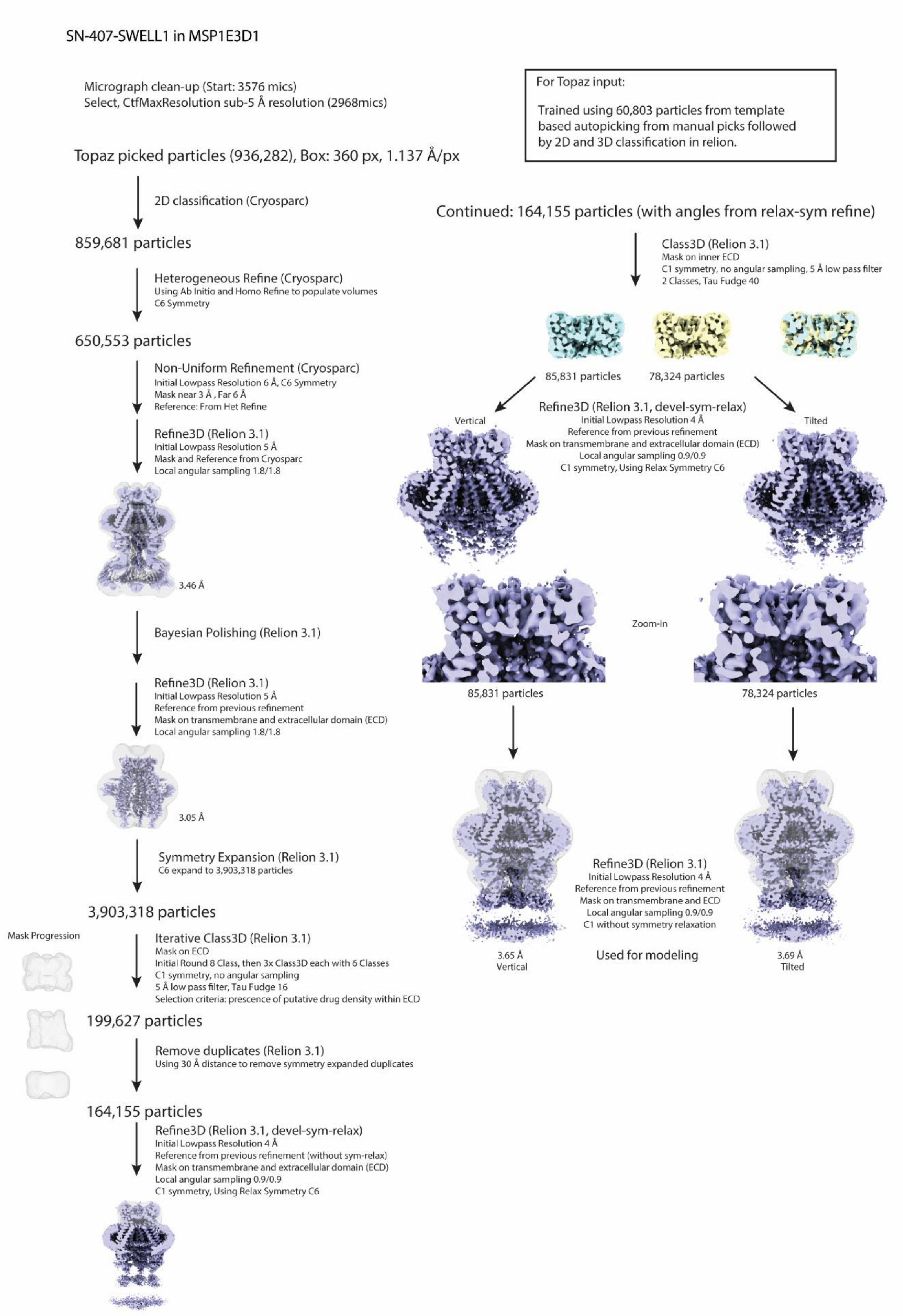
SN-407-SWELL1 in MSP1E3D1
Micrograph clean-up (Start: 3576 mics)
Select, CtfMaxResolution sub-5 Å resolution (2968mics)
For Topaz input:
Trained using 60,803 particles from template based autopicking from manual picks followed by 2D and 3D classification in relion.
Topaz picked particles (936,282), Box: 360 px, 1.137 Å/px
2D classification (Cryosparc)
859,681 particles
Heterogeneous Refine (Cryosparc)
Using Ab Initio and Homo Refine to populate volumes
C6 Symmetry
650,553 particles
Non-Uniform Refinement (Cryosparc)
Initial Lowpass Resolution 6 Å, C6 Symmetry
Mask near 3 Å , Far 6 Å
Reference: From Het Refine
Refine3D (Relion 3.1)
Initial Lowpass Resolution 5 Å
Mask and Reference from Cryosparc
Local angular sampling 1.8/1.8
3.46 Å
Bayesian Polishing (Relion 3.1)
Refine3D (Relion 3.1)
Initial Lowpass Resolution 5 Å
Reference from previous refinement
Mask on transmembrane and extracellular domain (ECD)
Local angular sampling 1.8/1.8
3.05 Å
Symmetry Expansion (Relion 3.1)
C6 expand to 3,903,318 particles
3,903,318 particles
Mask Progression
Iterative Class3D (Relion 3.1)
Mask on ECD
Initial Round 8 Class, then 3x Class3D each with 6 Classes
C1 symmetry, no angular sampling
5 Å low pass filter, Tau Fudge 16
Selection criteria: prescence of putative drug density within ECD
199,627 particles
Remove duplicates (Relion 3.1)
Using 30 Å distance to remove symmetry expanded duplicates
164,155 particles
Refine3D (Relion 3.1, devel-sym-relax)
Initial Lowpass Resolution 4 Å
Reference from previous refinement (without sym-relax)
Mask on transmembrane and extracellular domain (ECD)
Local angular sampling 0.9/0.9
C1 symmetry, Using Relax Symmetry C6
Continued: 164,155 particles (with angles from relax-sym refine)
Class3D (Relion 3.1)
Mask on inner ECD
C1 symmetry, no angular sampling, 5 Å low pass filter
2 Classes, Tau Fudge 40
85,831 particles
78,324 particles
Refine3D (Relion 3.1, devel-sym-relax)
Initial Lowpass Resolution 4 Å
Reference from previous refinement
Mask on transmembrane and extracellular domain (ECD)
Local angular sampling 0.9/0.9
C1 symmetry, Using Relax Symmetry C6
Vertical
Tilted
Zoom-in
85,831 particles
78,324 particles
Refine3D (Relion 3.1)
Initial Lowpass Resolution 4 Å
Reference from previous refinement
Mask on transmembrane and ECD
Local angular sampling 0.9/0.9
C1 without symmetry relaxation
Used for modeling
3.65 Å
Vertical
3.69 Å
Tilted

Supplementary Fig. 9. Cryo-EM processing pipeline for SN-407-SWELL1 in MSP1E3D1 lipid nanodiscs. Overview of Cryo-EM data processing pipeline in cryoSPARC and Relion. See Methods for additional details.

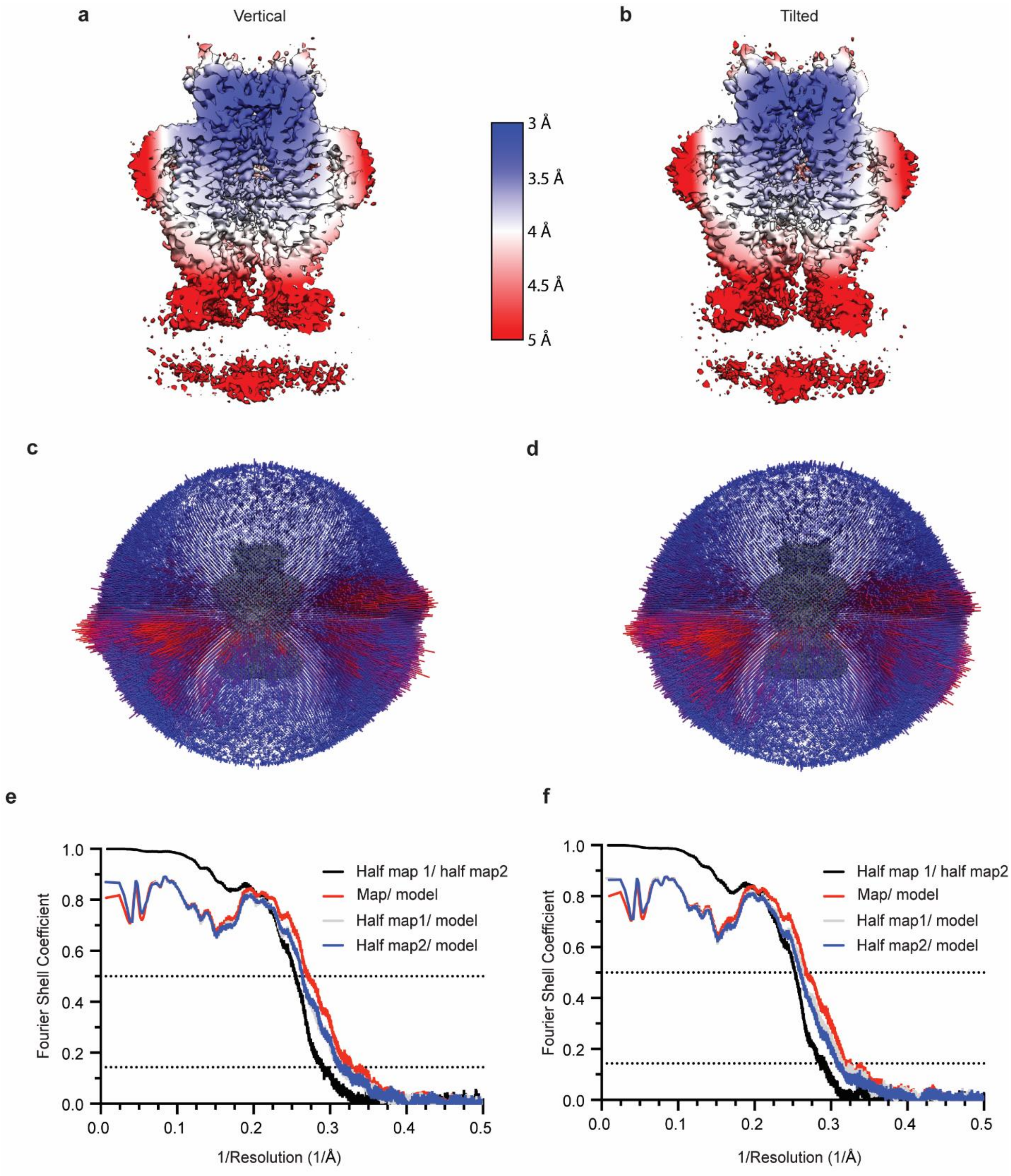

Supplementary Fig. 10. Cryo-EM validation for SN-407-SWELL1 in MSP1E3D1 lipid nanodiscs. a-b. Local resolution estimated in Relion colored as indicated on the final map for vertical (left) and tilted (right) drug density classes. **c-d.** Angular distribution of particles used in final refinement with maps for reference. **e-f.** Fourier Shell Correlation (FSC) relationships

between (black) the two unfiltered half-maps from refinement and used for calculating overall resolution at 0.143, (red) the final map and model, (gray) half-map one and model, and (blue) half-map and model.

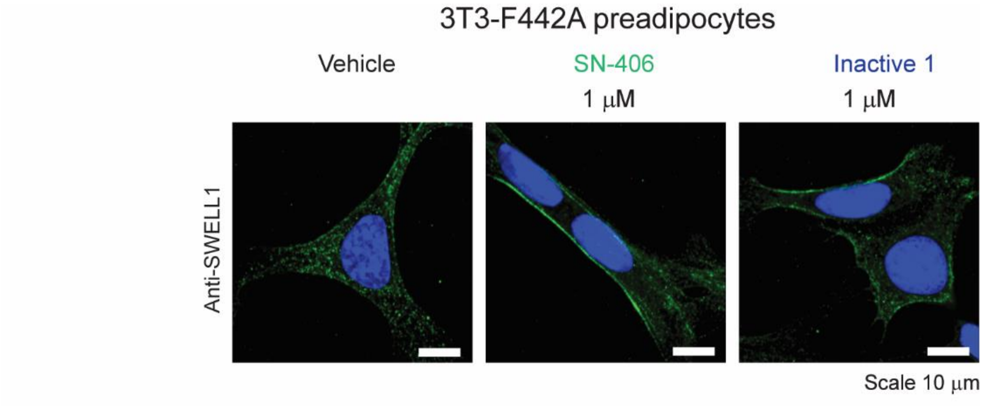

Supplementary Fig. S11. Active SN-401 congeners improve SWELL1 membrane localization at sub-micromolar concentrations. Representative immunostaining images demonstrating localization of endogenous SWELL1 in WT 3T3-F442A preadipocytes treated with either vehicle, SN-406 or Inactive 1 at 1 μM for 48h (Scale bar: 10 μm).

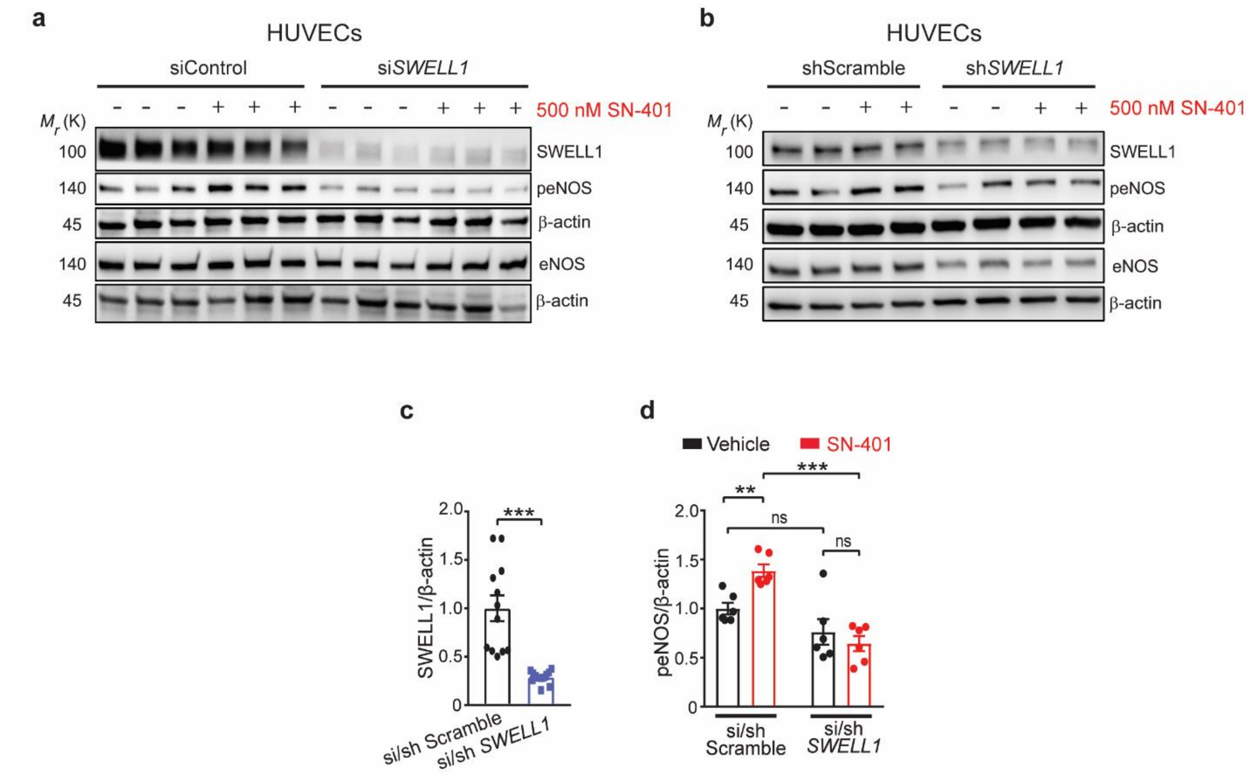

Supplementary Fig. S12. SN-401 mediated induction of peNOS activity in HUVECs is SWELL1 dependent. a. Western blots detecting SWELL1, $peNOS^{Ser1177}$, eNOS and β-actin in siControl and si*SWELL1* mediated knockdown in HUVEC cells treated with either vehicle or 500 nM SN-401 for 96 hours (n= 3 each). **b.** Western blots detecting SWELL1, $peNOS^{Ser1177}$, eNOS and β-actin in shScramble and sh*SWELL1* mediated knockdown in HUVEC cells treated with either vehicle or 500 nM SN-401 for 96 hours (n= 2 each). **c-d.** Densitometric ratios of SWELL1/β-actin (n=12 each) (**c**) and peNOS/β-actin (n=6 each) (**d**) combined from small interfering and short hairpin mediated *SWELL1* knockdown in **a** and **b**. Data are represented as mean ±SEM. Two-tailed unpaired t-test was used in **c** & **d.** *, ** and *** represents $p<0.05$, $p<0.01$ and $p<0.001$ respectively. ‘ns’ indicates the difference was not significant.

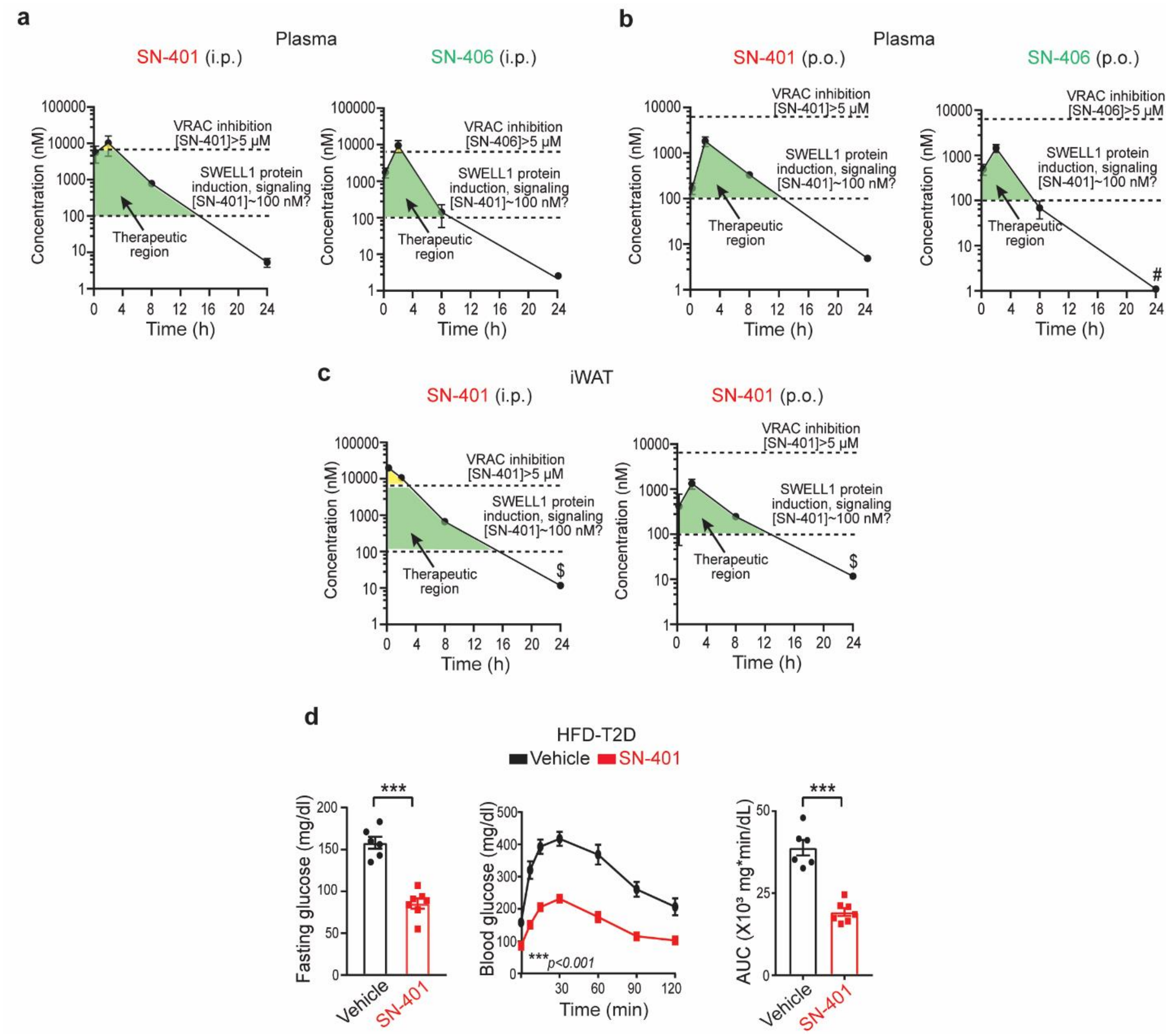

Supplementary Fig. S13. SN-401 and SN-406 *in vivo* pharmacokinetics and oral efficacy. **a-b.** SN-401 and SN-406 *in vivo* pharmacokinetics 5 mg/kg intraperitoneally (**a**) and by oral gavage (**b**) in plasma (n=3 mice for each time point, #-below detection limit of 1 nM). **c.** SN-401 *in vivo* pharmacokinetics 5 mg/kg intraperitoneally (i.p.) and by oral gavage (p.o.) in iWAT (n=3 mice for each time point, $-below detection limit of 11 nM). **d.** Fasting glucose levels, GTT and AUC of HFD-T2D mice (10 weeks HFD) treated with either vehicle (n = 6 males) or SN-401 (5 mg/kg p.o, n = 7 males) for 5 days. Data are represented as mean ±SEM. Two-tailed unpaired t-test was used in **d** for FG and AUC. Two-way ANOVA was used for GTT in **d**. Statistical significance is denoted by *, ** and *** representing $p<0.05$, $p<0.01$ and $p<0.001$ *respectively*.

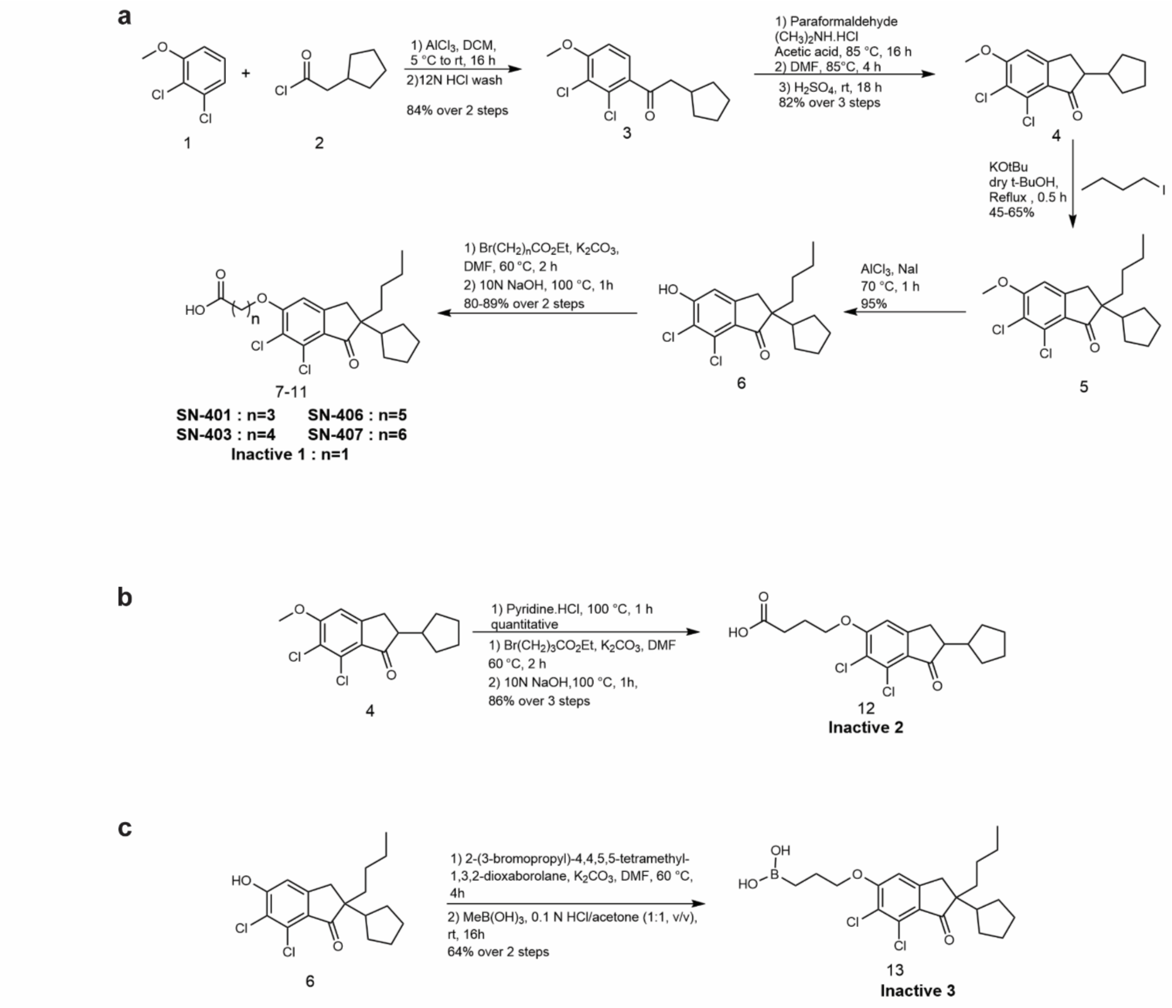

Supplementary Fig. S14. Scheme for chemical synthesis of SN-40X SWELL1 modulators and inactive compounds. a. Synthesis of SN-401, SN-403, SN-406, SN-407 and Inactive 1. **b**. Synthesis of Inactive 2. **c**. Synthesis of Inactive 3.

Supplementary Tables

Patient	Age (years)	Sex	BMI	Random Glucose (mg/dl)	Estimated Glucose (mg/dl)	HbA1c (%)
**Non-T2D**	44	F	26.8	151.8	NA	6.1
	57	M	28.7	144.3	NA	5.3
	24	F	32.2	234	NA	NA
**T2D**	46	F	35.9	262.4	NA	6.8
	37	F	38.1	253.8	NA	8.2
	51	M	35.59	NA	157	7.1

Supplementary Table. S1. Characteristics of patients from whom cadaveric non-T2D and T2D islets were obtained for β-cell patch-clamp studies in **Figure 1b&d**. NA, not available.

Patient	Age (years)	Sex	BMI	HbA1c (%)	Figure
**Non-T2D**	50	F	31.7	5.7	Fig. 1f
	61	M	19.6	5.9	Fig. 1f
	54	M	26.4	5.1	Fig. 1f
	31	M	26.2	5.3	Fig. 9d,e
	31	F	20.3	4.8	Fig. 9d,e
	66	M	25.6	4.9	Fig. 9d,e
**T2D**	62	M	25.9	10	Fig. 1f
	48	F	30.4	7.5	Fig. 1f
	54	F	24.4	7.2	Fig. 1f

Supplementary Table. S2. Characteristics of non-T2D and T2D patients from whom cadaveric islets were obtained to measure SWELL1 protein expression and GSIS assay.

Patient	Age (years)	Sex	BMI	Random Glucose (mg/dl)	Estimated Glucose (mg/dl)	HbA1c (%)
**Lean#**	52	M	27.56	97	111	5.5
	61	F	28.36	112	NA	5.5
**Obese non-T2D#**	38	F	55.10	88	117	5.7
	65	F	32.02	100	111	5.5
	51	F	48.80	97	114	5.6
**Obese-T2D**	41	F	52.31	148	151	6.9

Supplementary Table. S3. Characteristics of lean, non-T2D, and T2D bariatric surgery patients from whom primary adipocytes were isolated for patch-clamp studies **Figure 1h**. #Data from lean and obese non-T2D patients were reported previously in Zhang, Y *et al*. (2017).

Patient	Age (years)	Sex	BMI	Random Glucose (mg/dl)	Estimated Glucose (mg/dl)	HbA1c (%)
**Lean non-T2D**	47	F	24.85	97	111	5.5
**Obese non-T2D**	36	F	43.05	106	114	5.6
**Obese non-T2D**	49	M	59.43	119	126	6.0
**Obese non-T2D**	49	F	41.6	101 (fast)	NA	5.7
**Obese non-T2D**	52	F	49.8	89 (fast)	NA	5.9
**Obese non-T2D**	48	F	50.18	84	97	5.0
**Obese non-T2D**	50	F	38.81	100	117	5.7
**Obese non-T2D**	43	M	57.65	105	105	5.3
**Obese-T2D**	57	F	53.69	273	183	8.0
**Obese-T2D**	53	F	57.21	122 (fast)	NA	6.4
**Obese-T2D**	65	M	40.53	250	229	9.6
**Obese-T2D**	52	F	37.70	109	160	7.2

Supplementary Table. S4. Characteristics of lean, obese non-T2D, and obese T2D patients from whom adipose samples were obtained to measure SWELL1 protein expression levels in **Figure 1k**.

Figure	Group	Body weight	SEM	N	Group	Body weight	SEM	N	Significance
**Fig. 3c (GTT)**	Vehicle	41.2	1.59	7	SN-401	37	1.85	7	ns
**Fig. 3c (ITT)**	Vehicle	40.9	1.51	7	SN-401	36.2	1.74	7	ns
**Fig. 3e (KKA^{a})**	Pre SN-401	32.9	1.49	3	SN-401	30.9	1.96	3	ns
**Fig. 3e (KKA^{y})**	Pre SN-401	45.4	1.03	6	SN-401	42.5	1.01	6	**
**Fig. 3f (KKA^{a})**	Pre SN-401	30.7	2.61	3	SN-401	30.9	1.80	3	ns
**Fig. 3f (KKA^{y})**	Pre SN-401	42.6	0.73	6	SN-401	43.4	1.13	6	ns
**Fig. 3j**	Vehicle	23.9	0.62	6	SN-401	24.3	0.88	6	ns
**Fig. 3l (GTT)**	Vehicle	47.7	1.83	6	SN-401	46	2.28	6	ns
**Fig. 3l (ITT)**	Vehicle	48.2	1.86	6	SN-401	44.3	2.50	6	ns
**Fig. 4a**	Vehicle	38.8	1.0	7	SN-401	37.5	0.9	8	ns
**Fig. 4f**	Vehicle	51.1	1.68	6	SN-401	43.5	2.92	6	*
**Fig. 10a**	Inactive 1	38	0.86	5	SN-403	37.2	0.86	5	ns
**Fig. 10b**	Pre SN-406	47.8	0.74	5	SN-406	45.4	1.05	5	*
**Fig. 10c**	Inactive 1	44.5	0.91	7	SN-406	44.7	0.74	7	ns
**Fig. 10f**	Inactive 1	38.4	0.85	5	SN-407	38.7	1.11	6	ns
**Suppl. Fig. 4b GTT**	Vehicle	25.7	0.33	7	SN-401	25.2	0.27	7	ns
**Suppl. Fig. 4c ITT**	Vehicle	26	0.31	7	SN-401	25.4	0.31	7	ns
**Suppl. Fig. 13d**	Vehicle	40.3	0.77	6	SN-401	37.2	0.89	7	*

Supplementary Table S5. Average body weights of mice used for *in vivo* experiments in this study.

**Data collection**	**SWELL1/LRRC8A with SN-407 (Pose-1)**	**SWELL1/LRRC8A with SN-407 (Pose-2)**
PDB	7M17	7M19
EMDB	23614	23616
EMPIAR	10662	
Total movies #	3576	
Selected movies #	2968	
Magnification	36,000 x	
Voltage (KV)	200	
Electron exposure (e^-/Å^2)	51.59	
Frame #	50	
Defocus range (um)	-0.7 to -2.2	
Super resolution pixel size (Å^2)	0.5685	
Binned pixel size (Å^2)	1.137	
**Processing**		
Initial particle images (no.)	936,282	
Final particle images (no.)	85,831	78,324
Map resolution Masked (Å, FSC = 0.143)	3.65 (Relion)	3.69 (Relion)
Symmetry imposed	C1	C1
**Refinement**		
Model resolution (Å, FSC = 0.143 / FSC = 0.5)	3.06/3.72	3.15/3.75
Map-sharpening B factor (Å^2)	0	0
Composition		
Number of atoms	16039	16309

Number of protein residues	1878	1878
R.m.s. deviations		
Bond lengths (Å)	0.005	0.004
Bond angles (Å)	0.851	0.861
Validation		
MolProbity score	1.41	1.43
Clashscore	3.15	3.81
Ramachandran plot		
Favored (%)	95.66	96.15
Allowed (%)	4.34	3.85
Disallowed (%)	0	0
Rotamer outliers (%)	0	0.06

Supplementary Table. S6. Cryo-EM data collection, processing, refinement, and modeling data for SWELL1-SN-407 in MSP1E3D1 nanodiscs for vertical and tilted poses of SN-407.

PK parameters	SN-401			SN-406		
	**Oral**	**Intravenous**	**Intraperitoneal**	**Oral**	**Intravenous**	**Intraperitoneal**
**AUCinf (ng*h/mL)**	4682	5958	23030	3131	6532	18180
**Oral Bioavailability**	79%	NA	NA	48%	NA	NA
**C_{max} (ng/mL)**	781	5443	4367	660.7	15130	4300
**T-half (h)**	2.585	1.428	2.056	2.058	0.7689	1.809

Supplementary Table S7. SN-401 and SN-406 *in vivo* PK parameters.
