# Supplementary material for: Small molecule SWELL1 complex induction improves glycemic control and nonalcoholic fatty liver disease in murine Type 2 diabetes
Source: Nat Commun. 2022 Feb 10;13:784. doi: 10.1038/s41467-022-28435-0 (PMC8831520; doi:10.1038/s41467-022-28435-0)
Supplement: Supplementary file 3 — Description of Additional Supplementary Files [file 41467_2022_28435_MOESM3_ESM.docx]

Description of Additional Supplementary Files

Title: Supplementary Data

Description: Supplementary Data 1a. 1H spectrum for 3, Supplementary Data 1b. 1H spectrum for 4, Supplementary Data 1c. 1H spectrum for 5, Supplementary Data 1d. 1H spectrum for 6, Supplementary Data 1e. 1H spectrum for Inactive 1, Supplementary Data 1f. 1H spectrum for SN-401, Supplementary Data 1g. 1H spectrum for SN-403, Supplementary Data 1h. 1H spectrum for SN-406, Supplementary Data 1i. 1H spectrum for SN-407, Supplementary Data 1j. 1H spectrum for Inactive 2, Supplementary Data 1k. 1H spectrum for Inactive 3
