# Supplementary material for: Small molecule SWELL1 complex induction improves glycemic control and nonalcoholic fatty liver disease in murine Type 2 diabetes
Source: Nat Commun. 2022 Feb 10;13:784. doi: 10.1038/s41467-022-28435-0 (PMC8831520; doi:10.1038/s41467-022-28435-0)
Supplement: Supplementary file 4 — Supplementary Data [file 41467_2022_28435_MOESM4_ESM.pdf]

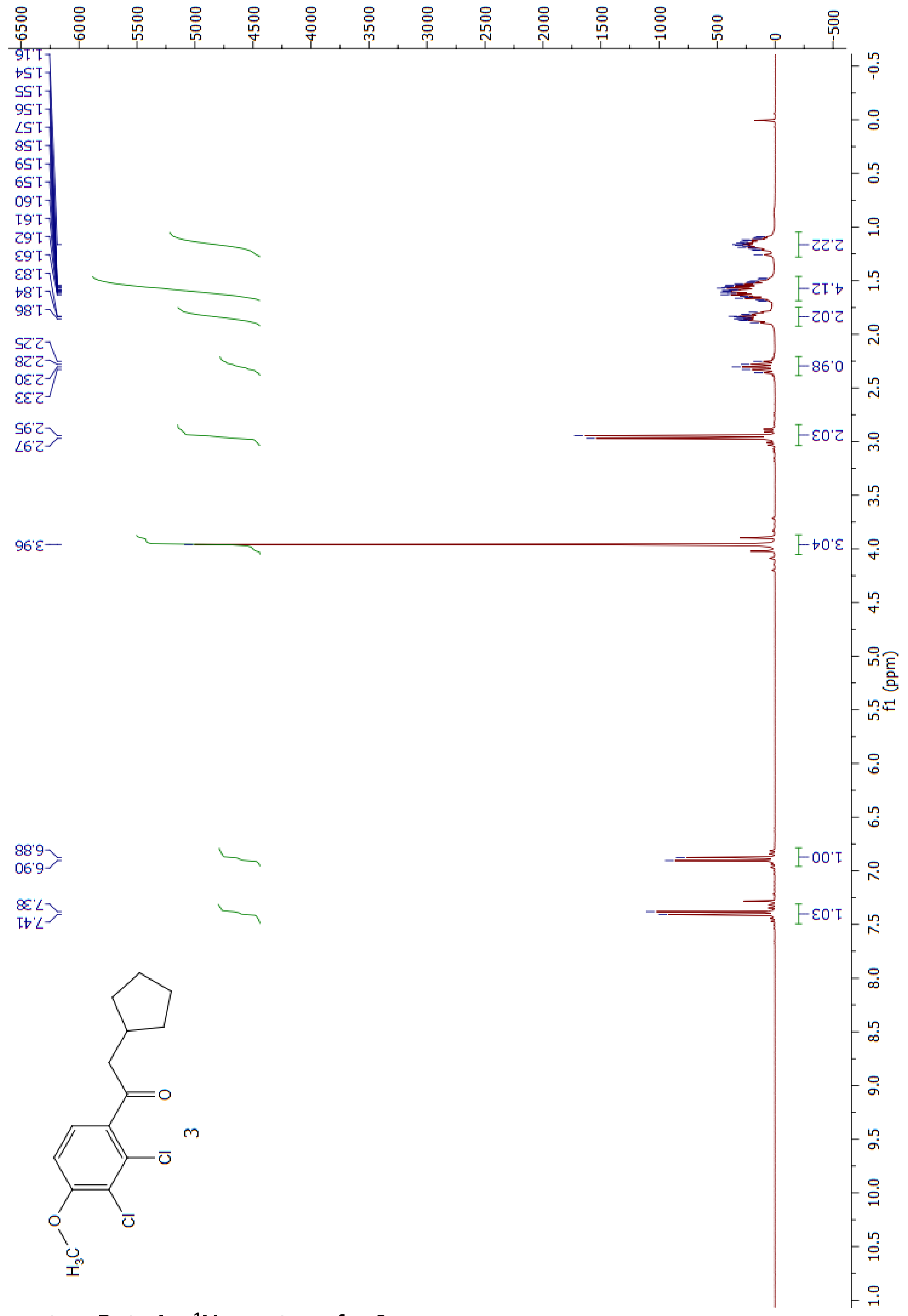

Supplementary Data 1a. ^{1}H spectrum for 3.

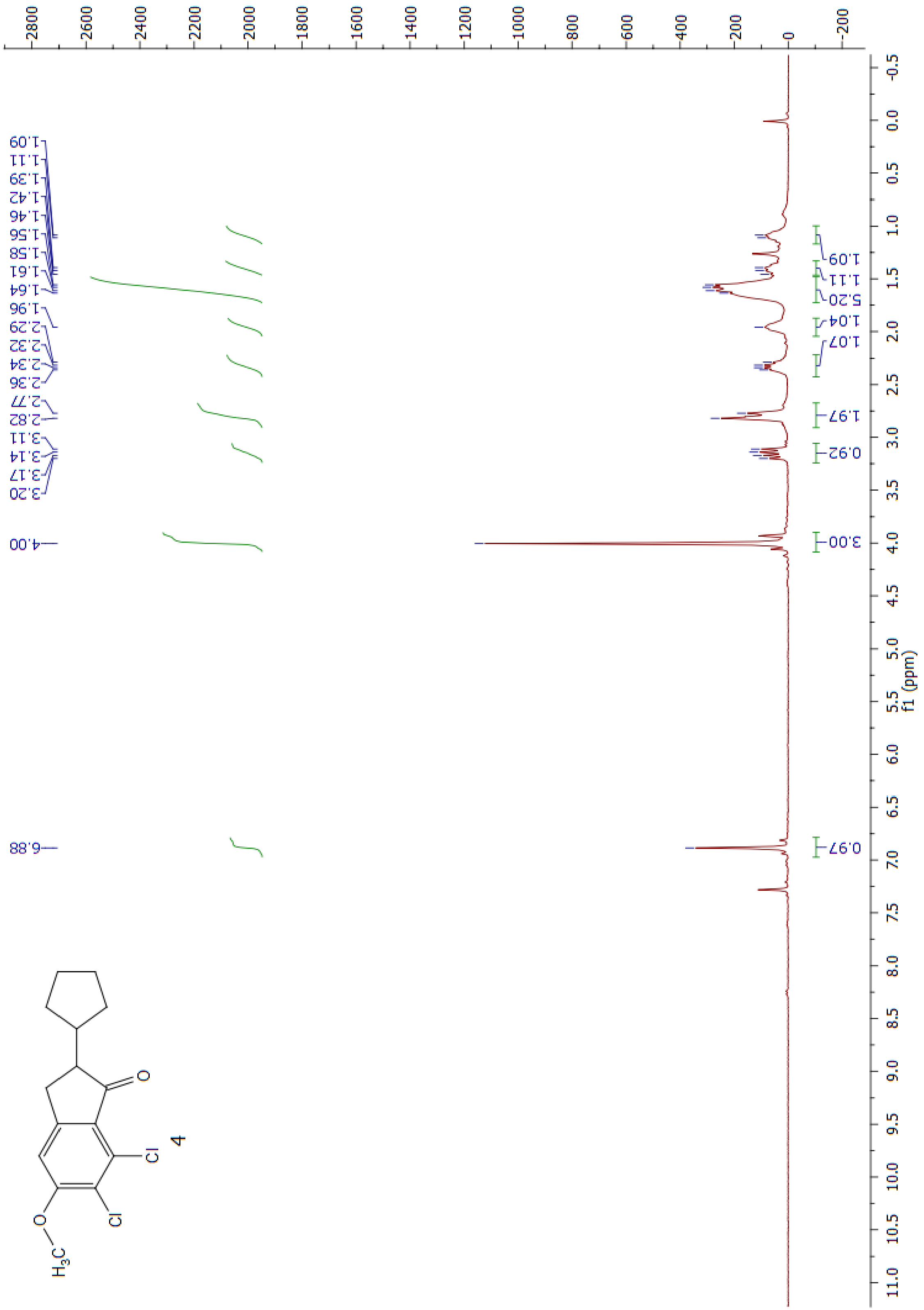

Supplementary Data 1b. ^{1}**H spectrum for 4.**

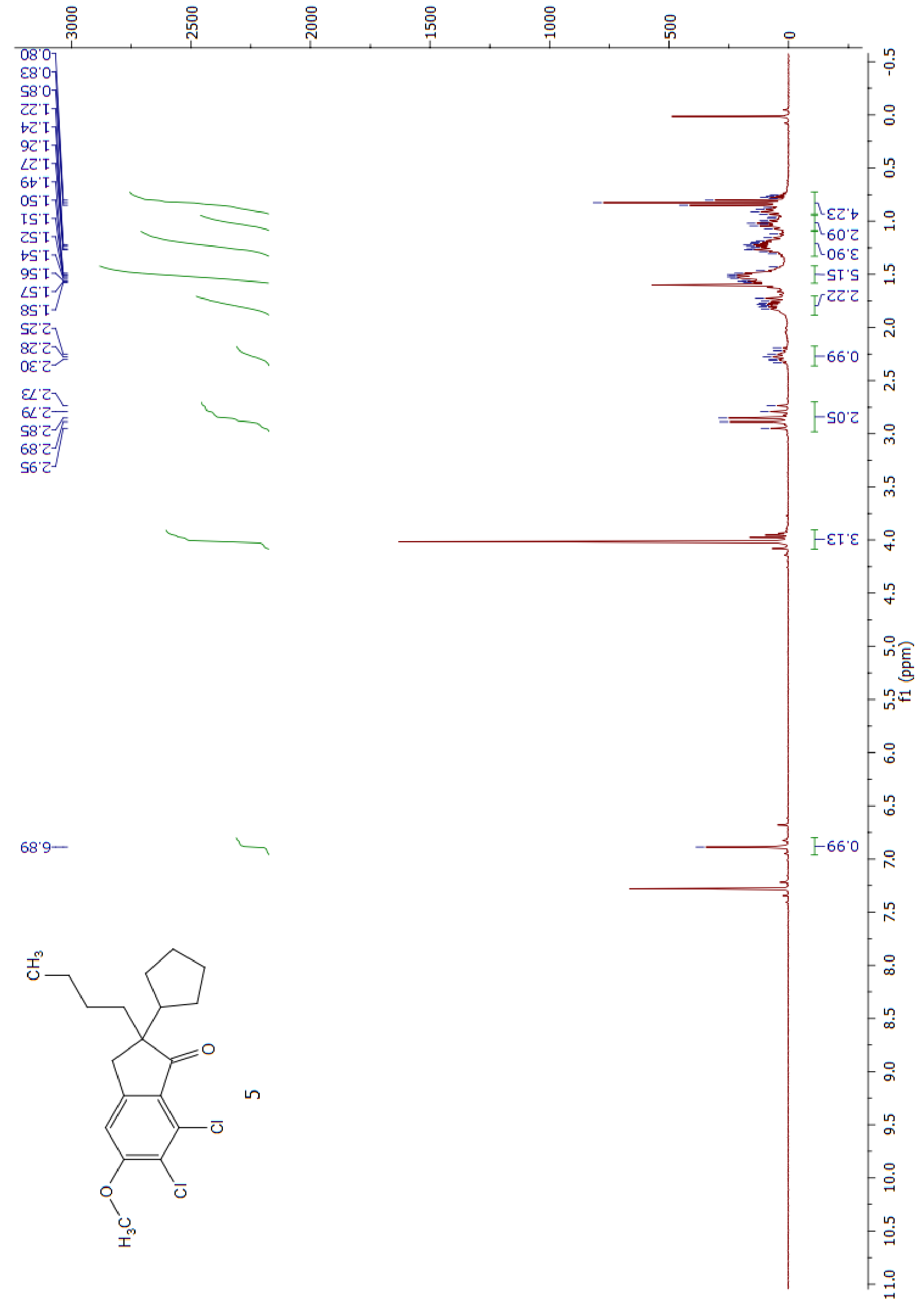

Supplementary Data 1c. ^{1}H spectrum for 5.

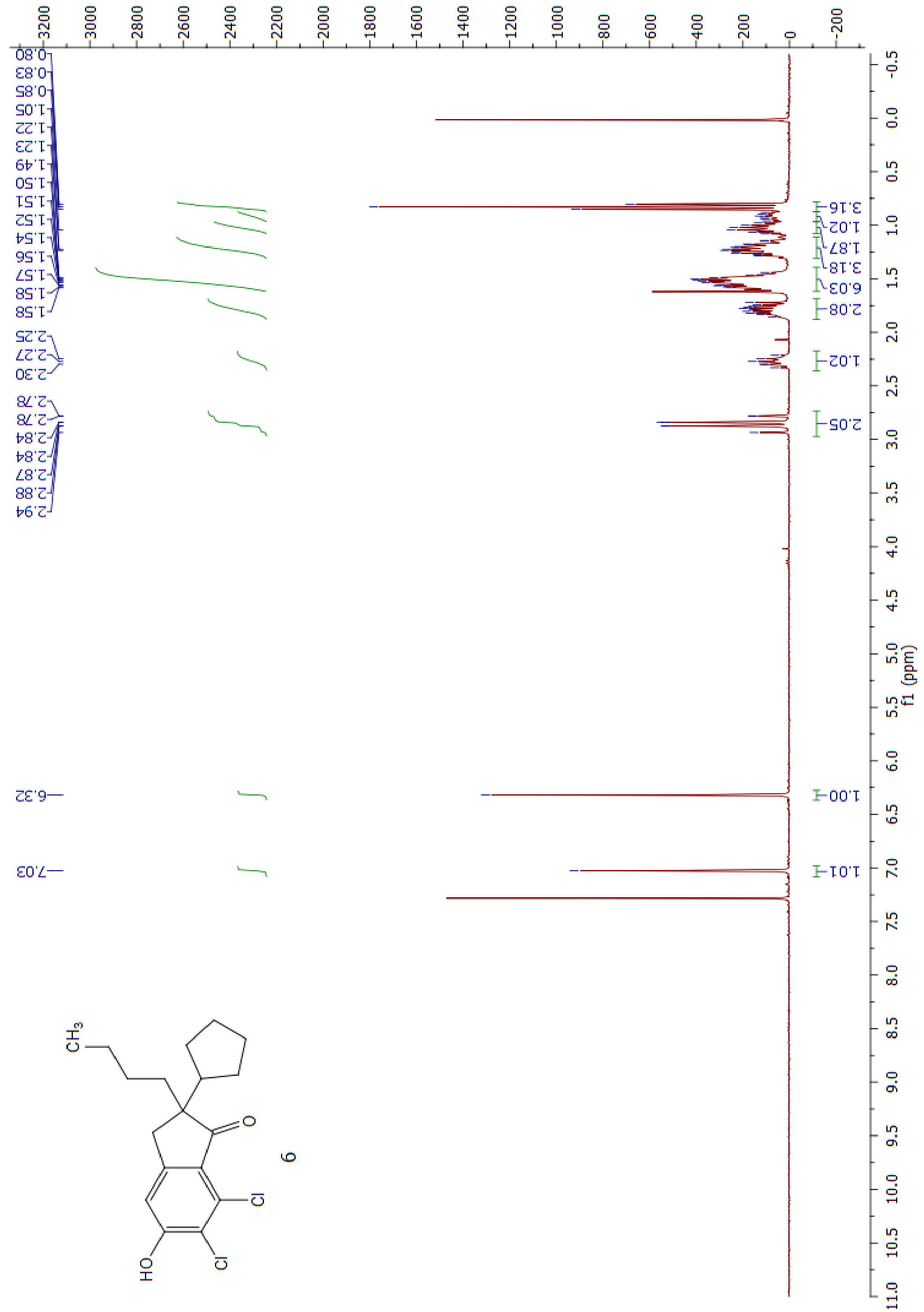

Supplementary Data 1d. ^{1}H spectrum for 6.

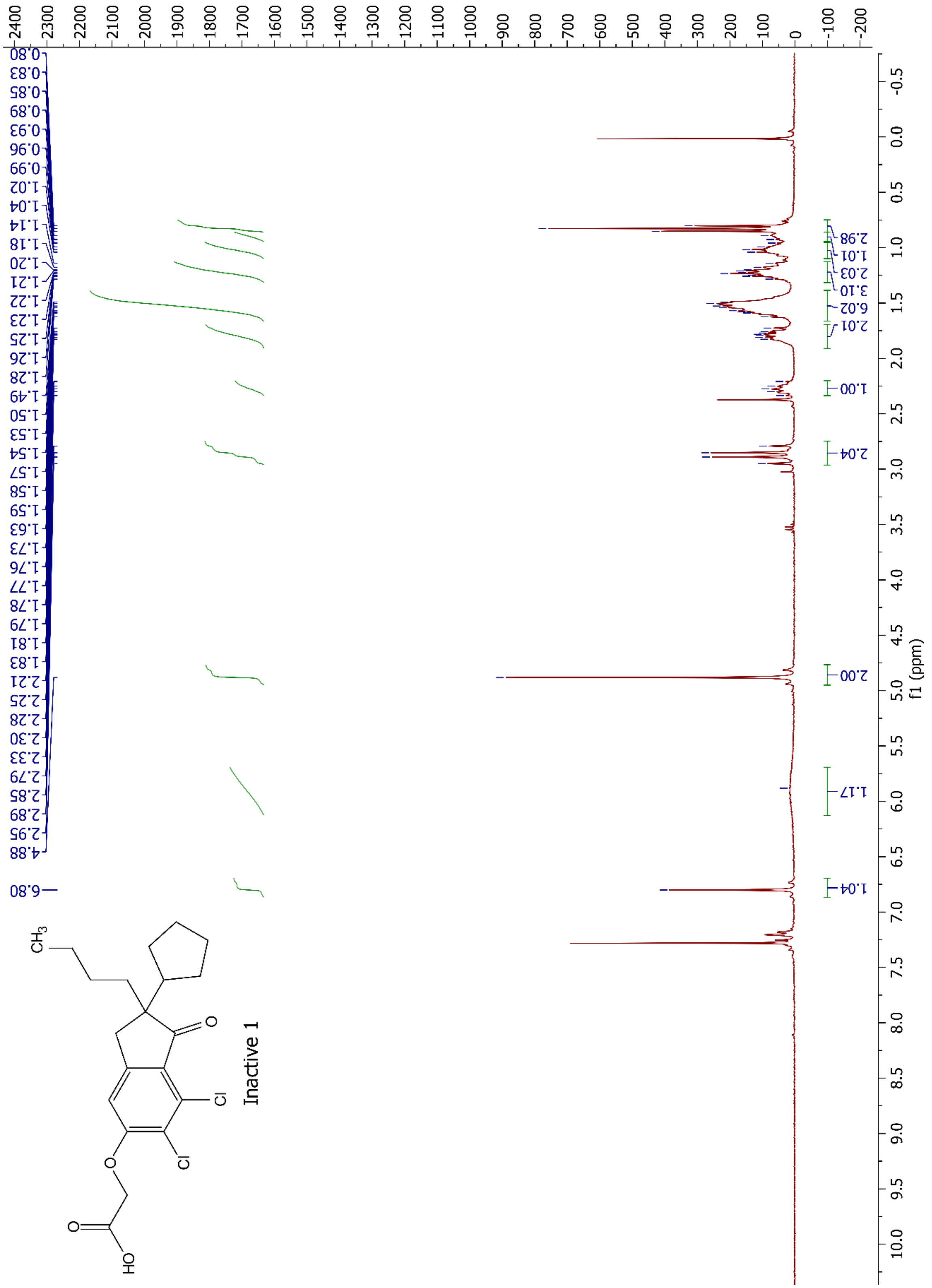

Supplementary Data 1e. ^{1}H spectrum for Inactive 1.

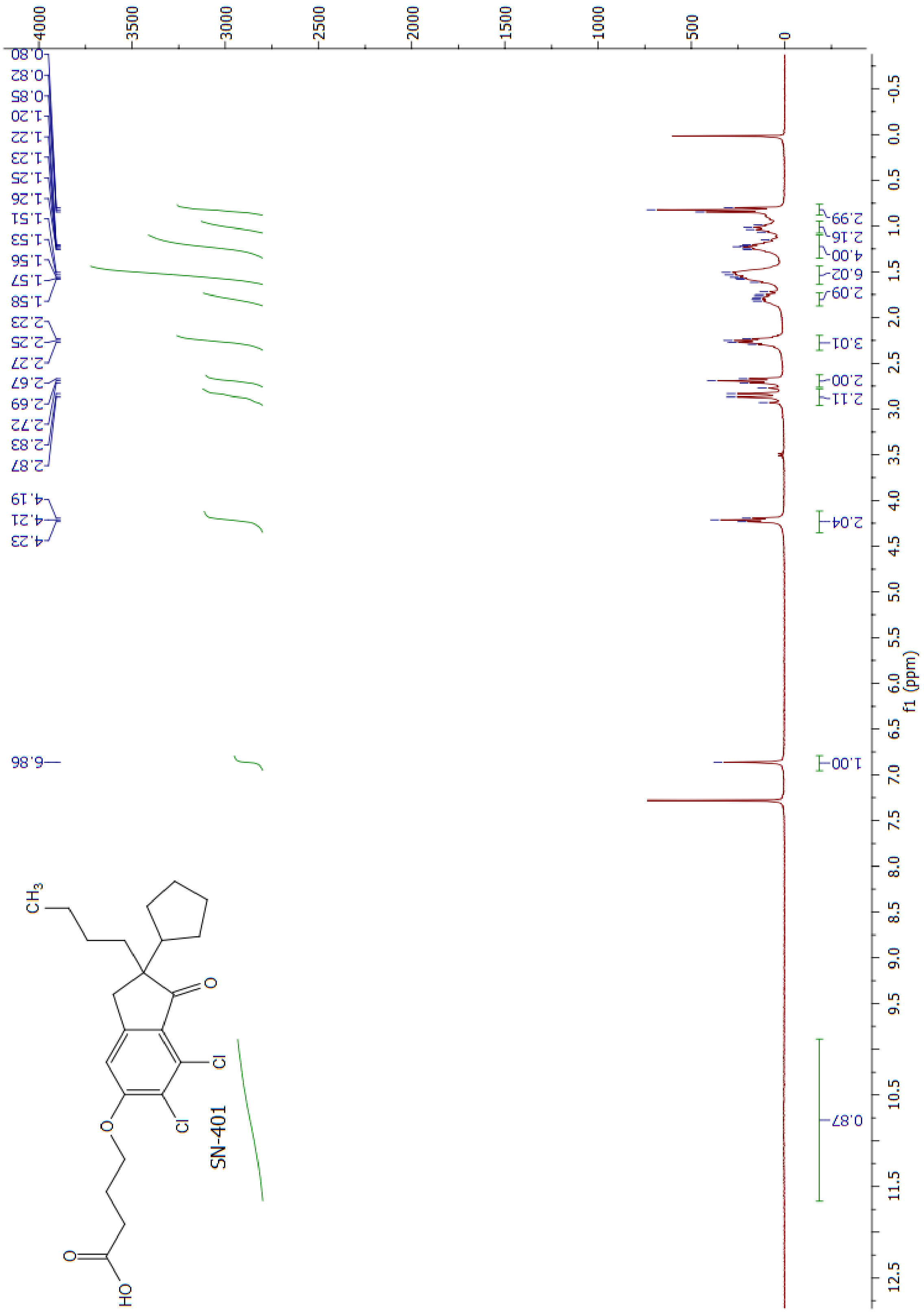

Supplementary Data 1f. ^{1}H spectrum for SN-401.

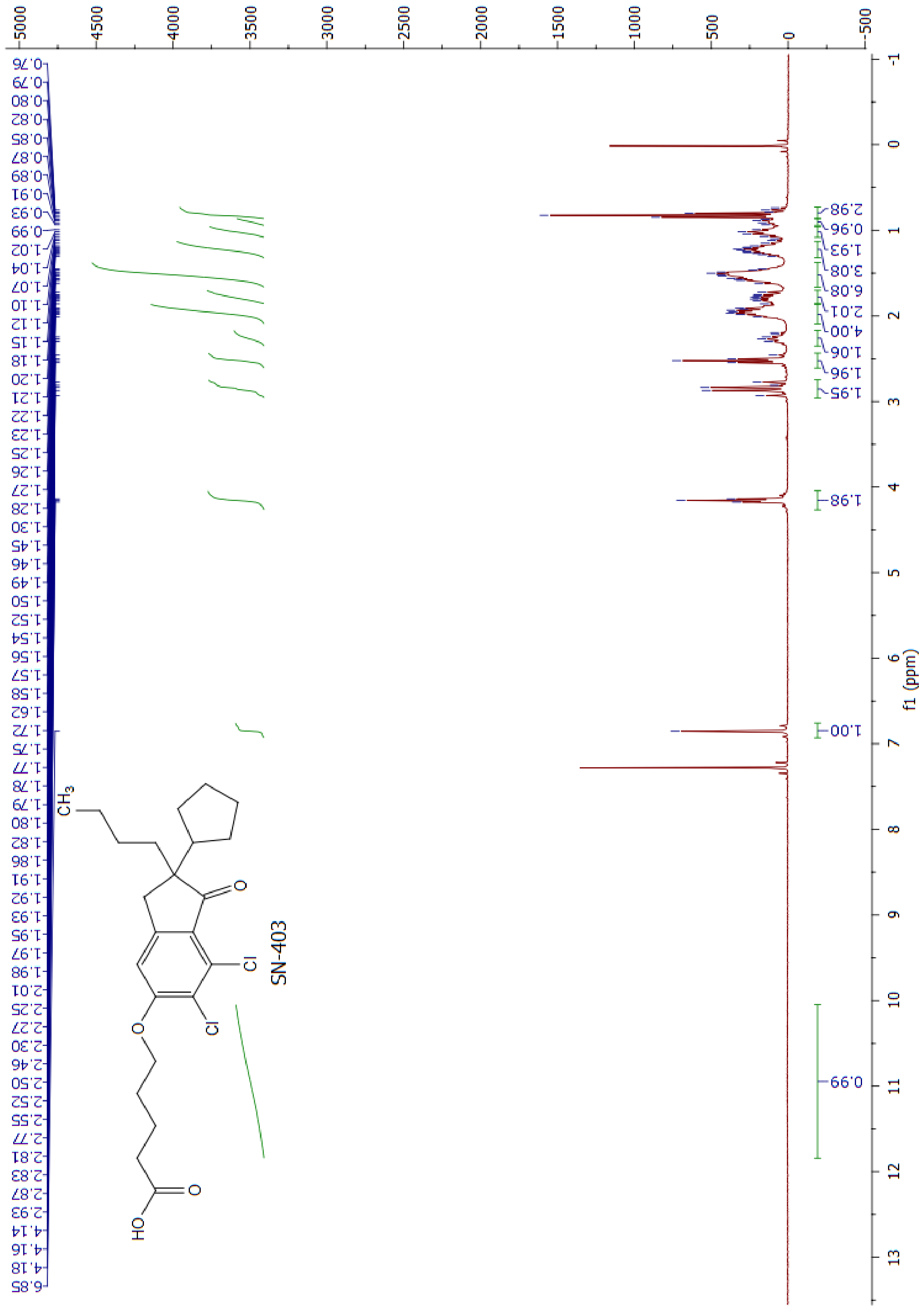

Supplementary Data 1g. ^{1}H spectrum for SN-403.

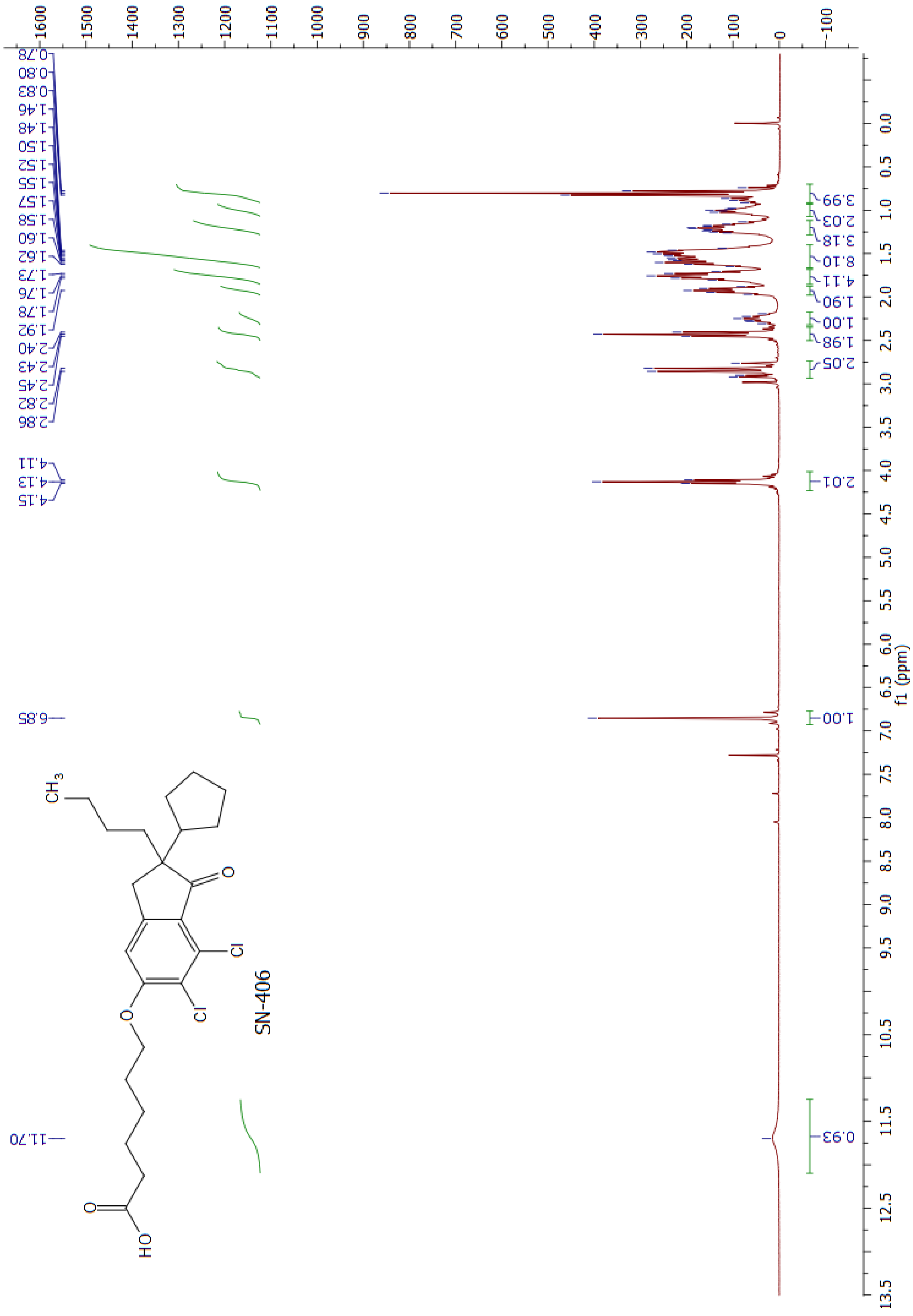

Supplementary Data 1h. ^{1}H spectrum for SN-406.

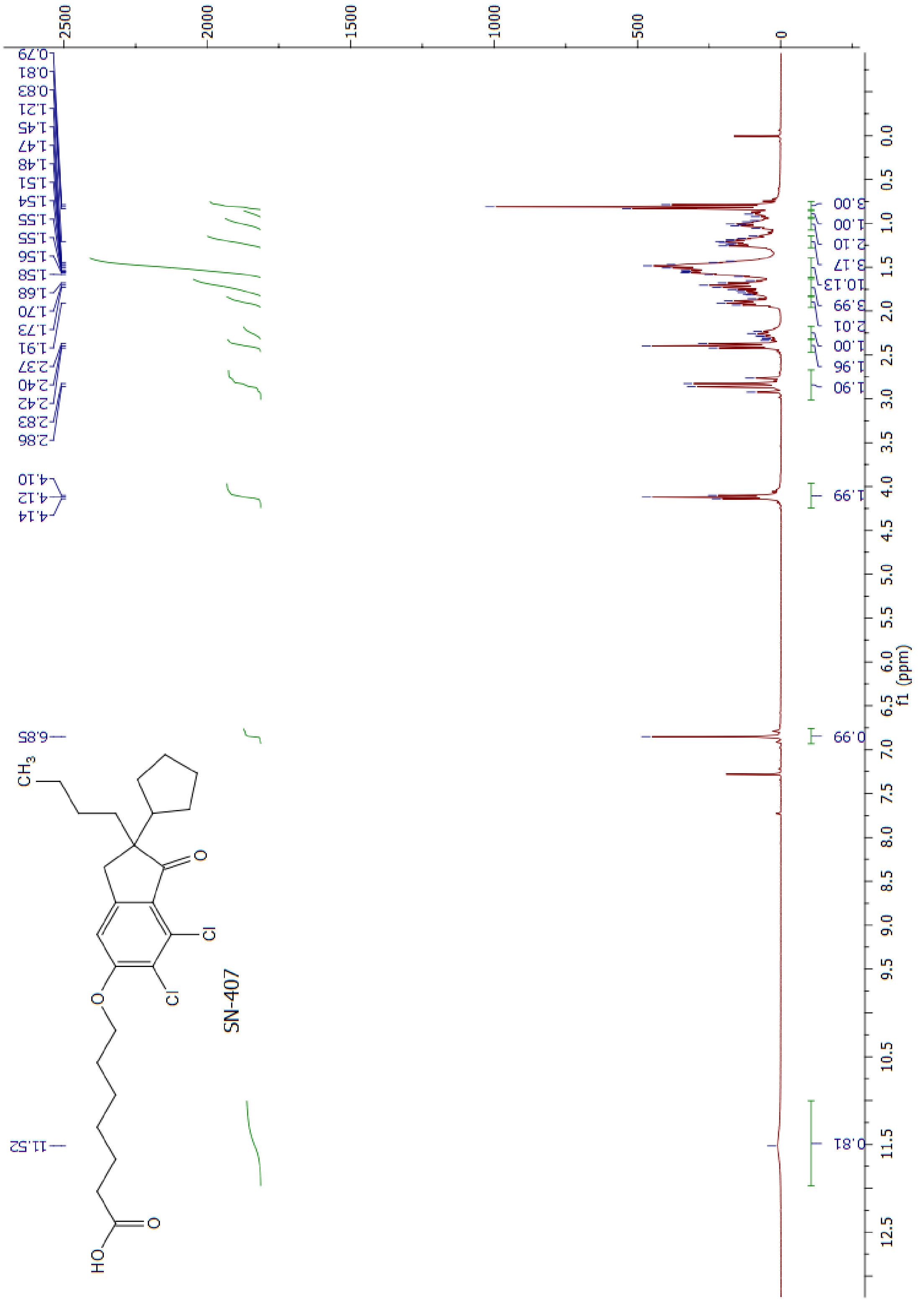

Supplementary Data 1i. ^{1}H spectrum for SN-407.

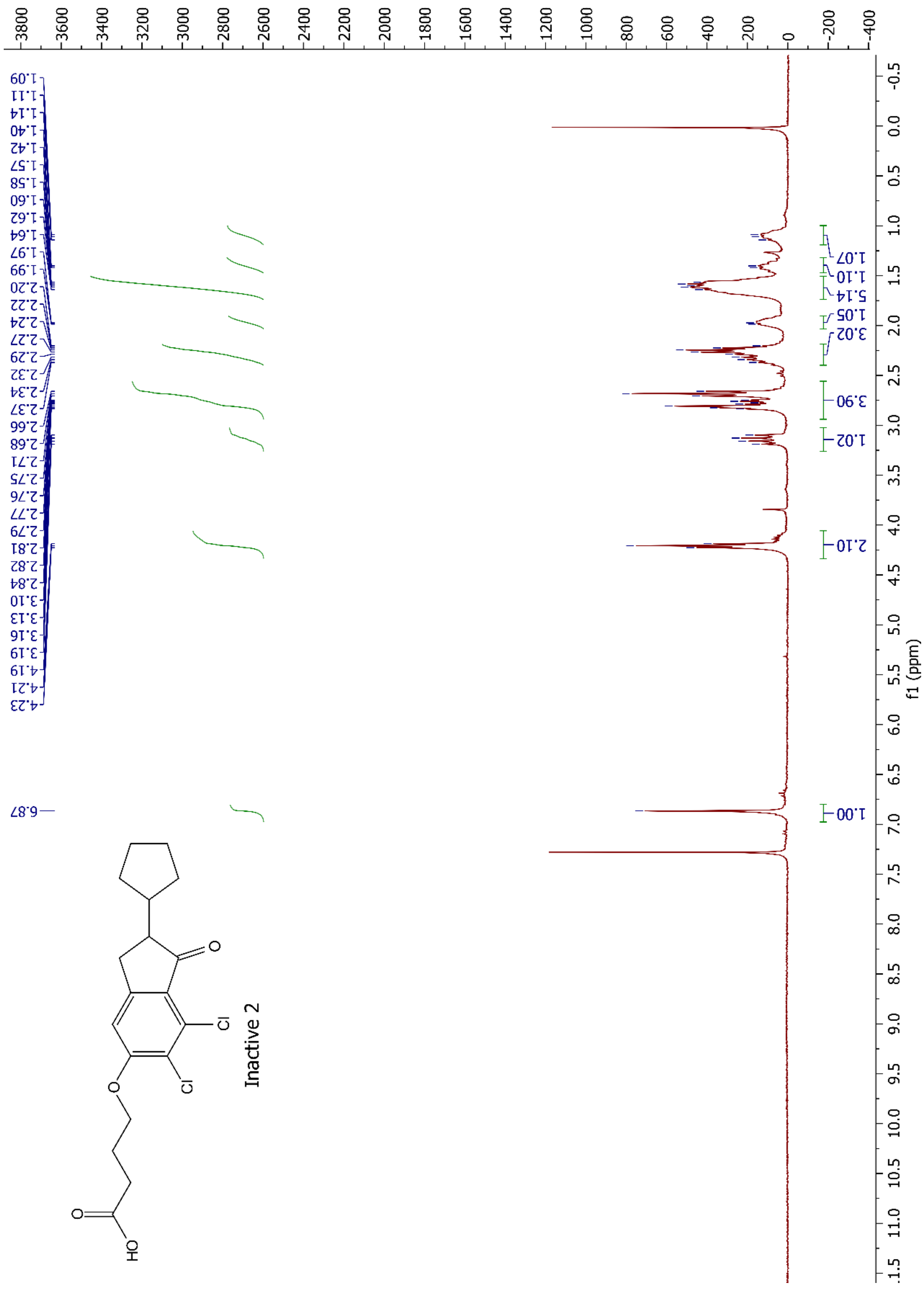

Supplementary Data 1j. ^{1}H spectrum for Inactive 2.

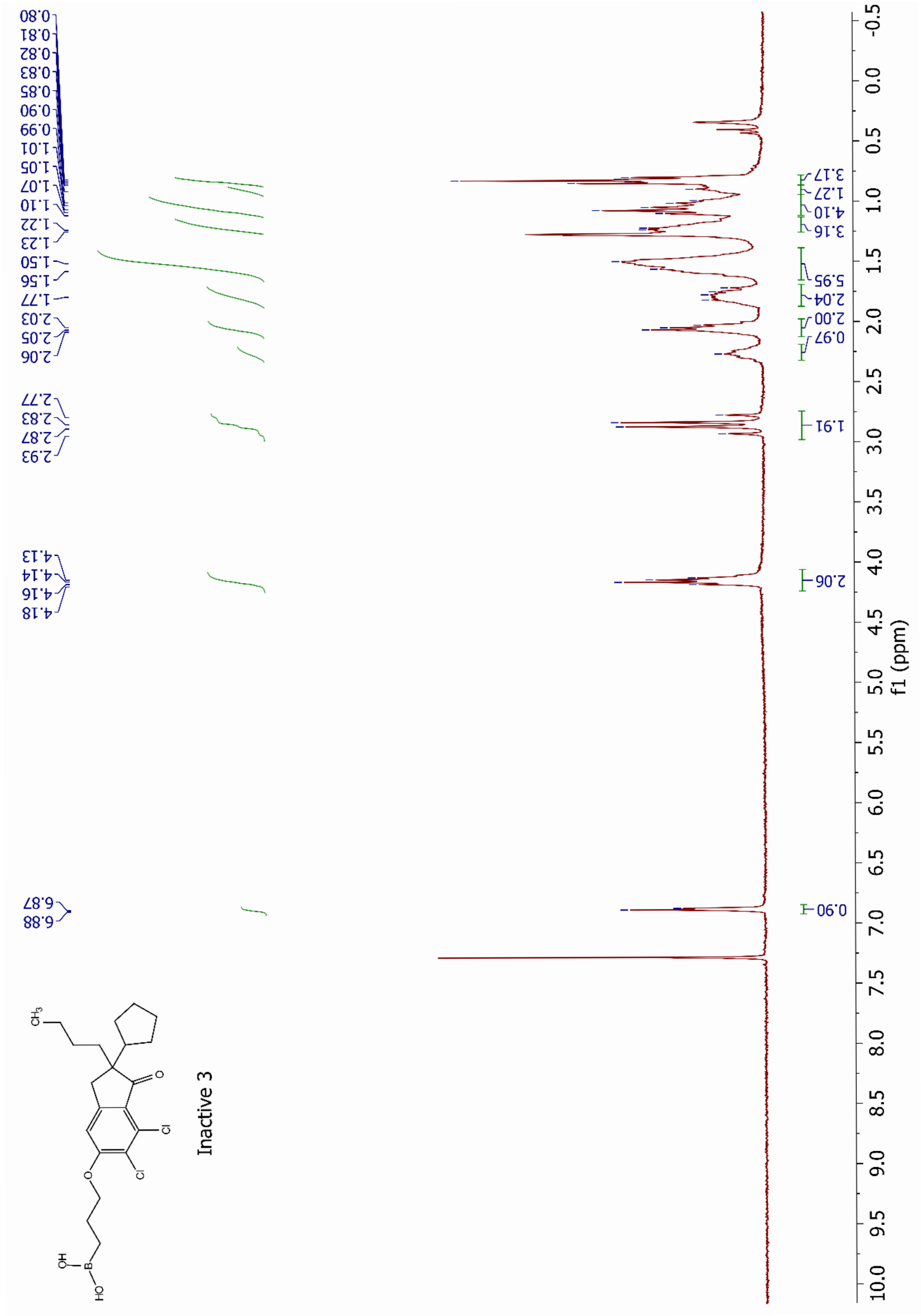

Supplementary Data 1k. ^{1}H spectrum for Inactive 3.
